# Spatiotemperal Dynamics of Osteoarthritis: Bridging Insights from Bench to Bedside

**DOI:** 10.14336/AD.2024.1538

**Published:** 2025-12-27

**Authors:** Xiwei Fan, Hong Xu, Indira Prasadam, Antonia Rujia Sun, Xiaoxin Wu, Ross Crawford, Yanping Wang, Xinzhan Mao

**Affiliations:** ^1^Department of Orthopaedic Surgery, The Second Xiangya Hospital of Central South University, Changsha, China.; ^2^Traumatic Orthopaedic Research Lab, The Second Xiangya Hospital of Central South University, Changsha, China.; ^3^Centre for Biomedical Technologies, Queensland University of Technology, Brisbane, Australia.; ^4^School of Mechanical, Medical & Process Engineering, Queensland University of Technology, Brisbane, Australia.; ^5^The Prince Charles Hospital, Brisbane, Australia.; ^6^Health Management Center, Xiangya Hospital, Central South University, Changsha, China.

**Keywords:** Aging, Degenerative diseases, Microenvironment, Osteoarthritis, Spatiotemporal analysis

## Abstract

Osteoarthritis (OA) is a multifaceted degenerative joint disorder affected by various risk factors such as age, mechanical stress, inflammation, and metabolic influences. These elements contribute to its diverse phenotypes and endotypes, underscoring the disease's inherent complexity. The involvement of multiple tissues and their interplay further complicates OA's investigation. The current limitations in spatial phenotyping technologies, coupled with the intricate web of multifactorial interactions, have hindered the discovery of reliable early diagnostic markers and the development of tailored therapeutic strategies. However, recent advances in spatiotemporal analysis have revolutionised researchers' capacity to explore OA's spatiotemporal dynamics. These advancements provide unprecedented insights into the disease's progression, revealing patient-specific clinical presentations, tissue and joint structure alterations, and microscopic to molecular changes in tissue cell populations and extracellular matrices. This paper summarises the latest developments in utilising state-of-the-art technologies for the deep phenotyping of OA's spatiotemporal variations, emphasising their critical role in elucidating OA's pathophysiology and how this can change clinical practice and advancing personalised treatment approaches, and finally lead to better clinical outcomes.

## 1. Introduction

Osteoarthritis (OA) is the most prevalent form of degenerative joint disease. Patients with OA frequently experience joint pain and limited mobility in multiple joints [1]. Additionally, weight-bearing joints affected by OA are also at significantly increased risk of other cormobilities, including falling and fracturing [2]. The latest epidemiological studies have found that the prevalence of OA accounts for over 7% of the world's population, with an estimated 527.81 million cases of OA worldwide [3]. Of these cases, 40% are symptomatic in patients with moderate to severe imaging. In the United States, more than 400,000 joint replacements are performed annually, and the cost of OA treatment accounts for 0.25% to 2.5% of the gross national product, and these data increase yearly [4]. In addition, OA is a major contributor to disability [5], thereby imposing a considerable burden on both individuals and society. The current management of OA has failed to stop or alleviate the disease progression, leaving joint replacement as the last choice, primarily due to the lack of methods that prevent OA progression, owing to a limited understanding of OA aetiology.

The progression of different OA phenotypes varies from several years to decades; therefore, tracking the dynamic factors influencing OA progression is crucial for a comprehensive understanding. Nevertheless, existing research in OA predominantly focuses on evaluating limited instances of osteoarthritic progression at specific time points. However, OA exhibits complex phenotypes and endotypes across different levels, and part of the truth may hinder a complete understanding of OA’s pathology and aetiology [6]. Fortunately, spatiotemporal variation has been primarily introduced to the epidemiology of OA to account for variations in incidence, timing, and distribution influenced by various factors. It refers to the dynamic changes occurring in OA progression's spatial and temporal dimensions, which involve changes from two aspects: spatial and temporal changes that are holistically integrated. The spatial changes matter due to the highly organised structure of the osteochondral unit, which includes different layers of cartilage, calcified cartilage zone, subchondral bone plate and cancellous bone, each reacting differently during OA progression [7]. The variability in disease progression timelines and the susceptibility of OA to diverse risk factors contribute to the heterogeneity of OA phenotypes, posing significant challenges to developing precision medicine approaches. This complexity partly explains the high failure rate of clinical trials to treat OA. Insight into spatiotemporal variation helps understand OA's epidemiological patterns [8, 9], disease burden, and healthcare needs across different times and places, guiding the optimal allocation of healthcare resources [10]. Advances in imaging, big data, artificial intelligence [11-13], and precision medicine have shifted the focus to OA's spatiotemporal dynamics, emphasising its complex, multi-stage progression influenced by genetic, metabolic, biomechanical, and environmental factors [14, 15]. Recent advancements in imaging, tissue analysis, molecular biology, bioinformatics, and artificial intelligence (AI) analytics have enhanced our ability to study these variations in detail, offering early and precise intervention opportunities [16]. For advanced-stage OA patients, these technologies enable more accurate identification of disease subtypes and phenotypes, improving patient care by facilitating precision treatment plans based on the evolution of joint structure and function, thus improving treatment outcomes and quality of life [17].

**Figure 1.**
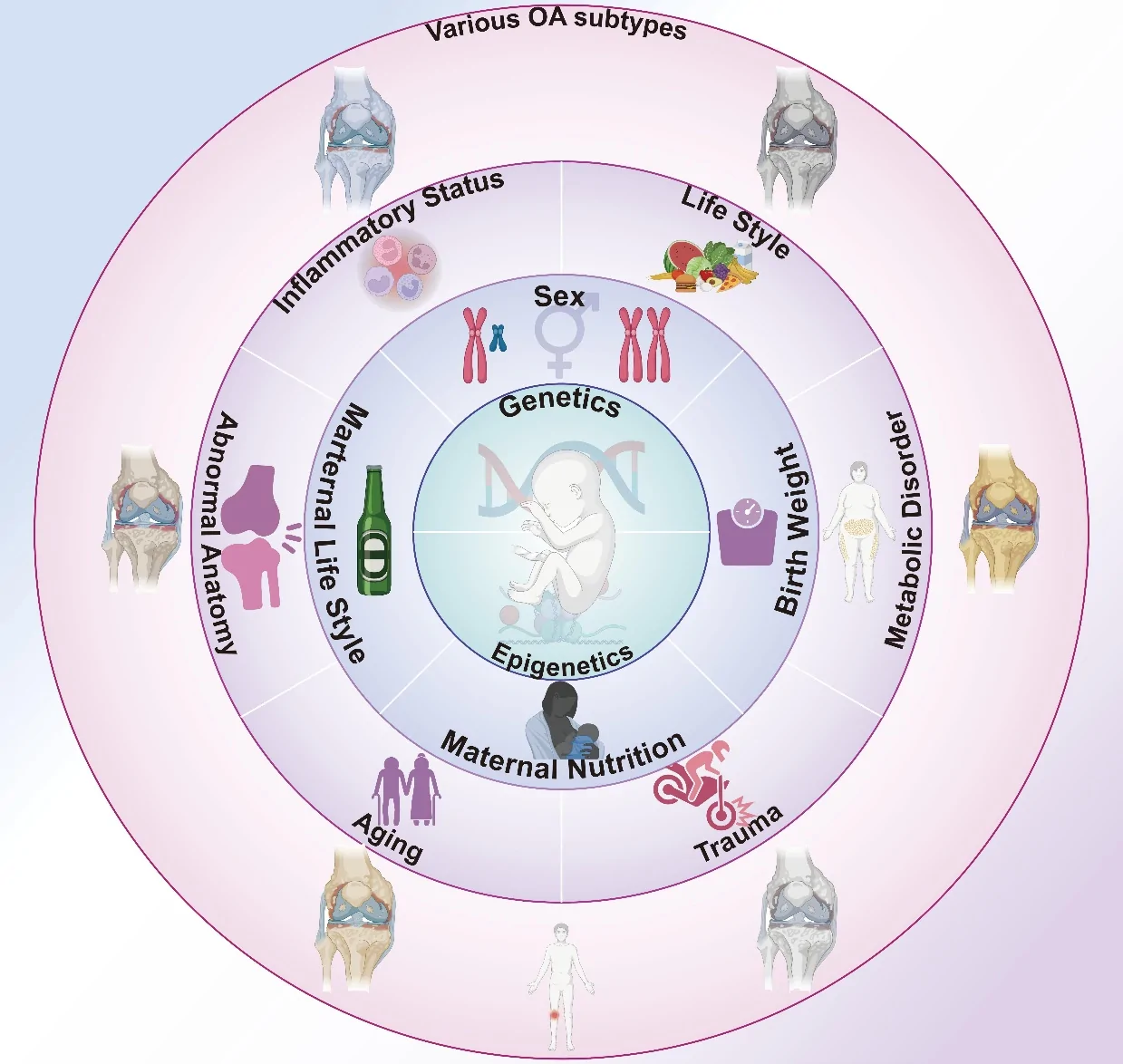
**Multiple factors influence the development of OA over the lifespan**. The complex interplay of genetic, environmental, and behavioural factors contributes to developing various OA phenotypes across age stages. Starting from embryonic development through to the elderly, genetic and epigenetic variations, environmental influences, nutritional factors from birth, and personal behaviours such as smoking and alcohol consumption weave together to impact joint health. Metabolic factors, abnormal knee anatomy, and trauma are highlighted as significant risk factors intensifying and influencing age. All the above risk factors highlight the complexity of addressing OA subtypes and a deep understanding from a holistic view. The figure was created with Biorender.com

Research on OA's aetiology and pathological transformations aims to uncover more effective treatments. Initially perceived as a wear-and-tear issue [18], recent research and advances reveal OA's complex genesis involving joint deterioration due to aging, stress, inflammation, and metabolic disorders. These lead to issues like synovitis and cartilage degeneration, accelerated by gene alterations and signalling pathway interactions [19-21]. As OA advances, cellular metabolism abnormalities in joint cartilage spark extensive inflammatory responses, exacerbating joint damage. Current clinical imaging techniques fail to detail these molecular and microstructural changes, highlighting the need for advanced spatial-temporal analysis for a comprehensive disease understanding [22, 23]. Emerging spatially resolved technologies [24, 25] have been validated to play pivotal roles in OA research, offering insights into disease phenotyping at various levels [8, 26]. These advanced strategies enable detailed molecular information through analyses like transcriptomics and proteomics, enhancing our understanding of OA's pathogenesis [27, 28]. Technologies like hyperspectral analysis allow non-invasive molecular detection in joint tissues, promising new diagnostic tools [8]. Although various studies have been published in this area, limited review papers have been collected to summarise the abovementioned advancements. This paper reviews the spatiotemporal developments in OA research from bench to bedside, aiming to inform precision treatment and prevention strategies (Fig. 1).

## 2. Spatiotemporal Development Trajectory in OA at The Macro Level

### 2.1 OA Changes from Temporal Perspective

#### 2.1.1 Developmental Risk Factors Induce OA Heterogeneity in Adults

The heritability of OA is influenced by a complex interaction of genetic, epigenetic, fetal, and environmental factors that contribute to its development and progression across the lifespan (Fig. 1). Compared to other chronic musculoskeletal disorders, such as rheumatoid arthritis (RA), spinal arthritis (SpA) psoriatic arthritis (PsA), myalgia and osteoporosis, OA has a relatively more significant heritability [29]. Fetal risk factors are essential in the development and progression of OA. Genetic predispositions, including genetic and epigenetic factors, are critical in OA progression [30]. Solid familial aggregation has been previously observed in OA [31], and recent Musculoskeletal Pain in Ullensaker Study (MUST) cohort and Norweigian OA Twin study (Nor-Twin) suggested that maternal inheritance are contributor to OA than paternal inheritance [32, 33]. Further, Magnusson et al. estimated the gender-specific genetic contributions to knee OA surgeries [34] using the largest twin registry worldwide, and it was discovered that knee OA surgery had a heritability of 53%. Additionally, the genetic contribution in males varied with body mass index (BMI) and age.

Genetic polymorphisms can predispose individuals to OA. Genetic variations related to cartilage structure, joint formation, and inflammation can be inherited, raising the risk of contracting OA later in life. The main genes associated with OA onset include Pattern Recognition Receptors, Interleukin 1 (IL-1), Interleukin 6 (IL-6), Matrix Metalloproteinases (MMPs), A Disintegrin and Metalloprotease with Thrombospondin Type I Motifs (ADAMTS), Intelectin 1 (ITLN1), Active Chemokine C-Motif Ligand 2 (XCL2) and DOT1 like histone lysine methyltransferase (DOT1L) [35].

Meanwhile, maternal exposure to certain environmental factors can lead to epigenetic modifications, which may affect gene expression related to cartilage health and inflammation, thereby increasing OA susceptibility. Epigenetic modifications are vital in regulating gene expression, including deoxyribonucleic acid (DNA) methylation, histone changes, and chromatin remodelling. Ribonucleic acid (RNA) epigenetics contributes significantly to this regulatory process [36]. Maternal health and exposure to environmental risk factors are also crucial for OA risk in later life. This process's main epigenetic regulatory factors include DNA methylation, histone modifications, and non-coding RNA [37]. Maternal smoking and alcohol use during pregnancy further exacerbate these risks. The study verified that maternal smoking could impair fetal growth, leading to lower bone mass and joint abnormalities, thereby increasing the risk of OA via the transforming growth factor-beta (TGF-β) signalling pathway [38]. Excessive alcohol intake during pregnancy can also affect fetal development, leading to skeletal abnormalities and a higher likelihood of OA via the IGF-1 signalling pathway [39]. Another study explored the long-term effects of early-life exposure to hyperglycaemia on OA susceptibility [40], and the mice model showed that early hyperglycaemic exposure leads to increased OA risk in adulthood primarily due to persistent low Sirtuin 3 (Sirt) expression, which disrupts mitochondrial function and mitophagy in chondrocytes. Enhancing Sirt3 expression or using Sirt3 agonists like Honokiol was shown to mitigate these effects and slow OA progression, which suggests that gestational diabetes may increase OA risk through epigenetic changes.

Up to date, evidence shows that poor or excessive maternal nutrition will lead to a newborn’s OA in later life. Poor maternal nutrition, such as insufficient nutrition during pregnancy, can have an impact on fetal growth and development, thereby increasing the likelihood of OA in a newborn's later life. By programmatic alterations of the hypothalamic-pituitary-adrenal (HPA) axis-related neuroendocrine metabolism, low birth weight can be induced, along with changes in glucose and lipid metabolism, and finally contribute to the poor-quality state of articular cartilage [41]. On the contrary, maternal obesity in pregnant women can also lead to fetal macrosomia (large birth weight), which is associated with increased mechanical stress on developing joints and a higher risk of OA. Gestational diabetes can also result in larger fetal size and altered fetal metabolism, increasing the risk of joint abnormalities and OA later in life. Meanwhile, a high-fat diet (HFD) in parents and grandparents affects metabolic and skeletal health, systemic inflammation, and the risk of OA in two generations of mice offspring [42]. Researchers examined the metabolic profiles and OA susceptibility in first- and second-generation mice whose parents were fed high-fat or low-fat diets, and they found that parental obesity and high-fat diets negatively impact the musculoskeletal health of subsequent generations.

Meanwhile, preterm birth, low birth weight and growth patterns (like childhood obesity) can significantly influence the risk of developing joint disorders and OA. It was believed that babies born with low birth weight may have underdeveloped cartilage and hip bone abnormalities, which increases their susceptibility to hip OA and other joint disorders [43]. Earlier studies used dogs as the model animals for the longitudinal study, and they observed that dog body weight could alter hip development, which may result in early-onset hip OA [44]. This was supported by another meta-analysis [43], which deduced that low birth weight and preterm birth are associated with abnormal hip morphology and an increased risk of hip OA. No association between birth weight and OA was found. However, Zengfeng et al. recently used Mendelian randomisation to assess birth weight, childhood obesity, and OA occurrence in adults using the Genetics of OA Consortium (n = 826,690). The results showed that childhood obesity has shown a causal relationship with OA in weight-bearing joints, suggesting a general link between obesity and OA [45]. Another study from the National Survey of Health and Development (NSHD) (n=2989) also discovered that the changes in Body Mass Index (BMI) during childhood among women and the changes in BMI during adolescence in men were likewise positively correlated with knee OA at the age of 53. [46]. The risk of high BMI accumulates with adulthood, which emphatically accentuates the crucial significance of weight control spanning an entire lifetime as a primary preventive measure for knee OA.

Other developmental factors are also worth careful consideration. Abnormal fetal positioning, such as breech presentation, can affect joint development and alignment, increasing the likelihood of joint abnormalities, such as developmental dysplasia of the hip and further inducing secondary OA [47]. In the UK Biobank, early menarche was also associated with a higher risk of OA [48]. In summary, developmental risk factors can lead to impaired joint development, altered biomechanics, and a pro-inflammatory state, increasing the risk of OA later in life. Understanding these mechanisms highlights the importance of maternal health and prenatal care in preventing OA and improving long-term joint health.

#### 2.1.2 Longitudinal Study, Big Data, and Temporal Change of OA

Several databases have been established to track the OA progression temporally to monitor degenerative diseases, including OA. Famous databases include Osteoarthritis Initiative (OAI) [49, 50], UK Biobank [51], Multicenter Osteoarthritis Study (MOST) [52, 53], Framingham OA Study [54], Chingford General Population Survey [55], Rotterdam Study (ERGO) [56], Cohort Hip and Cohort Knee (CHECK) Study [57], Applied Public-Private Research enabling OA Clinical Headway funded by the Innovative Medicines Initiative (IMI-APPROACH) [58], Johnston County OA Project [59], Hertfordshire Cohort Study [60, 61], Tasmanian Older Adult Cohort Study (TASOAC) [62] and Netherlands Epidemiology of Obesity (NEO) Study [63] (Table 1). OAI identified genetic, biochemical, and imaging biomarkers that predict OA progression and established associated risk factors [64]. The UK Biobank has provided large-scale genomic data pinpointing genetic variants linked to OA and establishing connections between OA and cardiovascular diseases [49]. MOST explored risk factors such as age, obesity, and joint injury and assessed disease progression through imaging techniques [53]. The Framingham OA Study revealed a correlation between obesity and knee OA and identified both genetic predispositions and lifestyle factors influencing the disease. The Chingford General Population Survey examined the natural history and risk factors of OA, emphasising the impact of estrogen and menopause on the condition [65]. ERGO discovered associations between OA and metabolic syndrome, cardiovascular diseases, and genetic markers [56]. The CHECK study provided longitudinal data on early OA, identifying early symptoms and risk factors that predict disease progression [66]. IMI-APPROACH [58] identifies novel OA phenotypes, validates prediction models for OA progression and develops sensitive markers for OA progression [67, 68]. The Johnston County OA Project highlighted racial and ethnic disparities in OA prevalence and progression and explored environmental influences on the disease [69, 70]. The Hertfordshire Cohort Study linked early-life factors to later-life OA, underscoring the roles of nutrition and physical activity [60, 71]. The TASOAC assessed genetic and environmental risk factors for OA, finding strong associations with bone health and physical activity [72, 73]. Finally, the Netherlands' NEO Study examined the impact of obesity on OA, linking measures of adiposity with joint damage and pain [74]. The above longitudinal studies offered insights into the disease's progression. Magnetic Resonance Imaging (MRI) was particularly effective at detecting early cartilage changes and bone marrow edema, often correlated with other clinical manifestations [75].

**Table 1:** Overview of Major Longitudinal Studies on OA.

Database	Description	Data Type	Key findings	Study Duration	Sample Size
**OAI [49, 50]**	A multi-centre, longitudinal, prospective observational study of knee OA.	Clinical evaluation, biospecimens, MRI, X-rays, radiographs	Identified genetic, biochemical, and imaging biomarkers for OA progression; established risk factors [64].	10 years (started in 2004)	Approximately 4,796 participants
**UK Biobank [51]**	A large-scale biomedical database with detailed imaging and health data.	Genetic data, health records, imaging data	Large-scale genomic data identified genetic variants associated with OA and linked OA to cardiovascular diseases [49].	Ongoing since 2006	Over 500,000 participants
**MOST [52, 53]**	A longitudinal study focused on the risk factors and prevention of knee OA.	Clinical assessments, X-rays, MRI	Identified risk factors, including age, obesity, and joint injury; assessed disease progression using imaging [53].	15 years (started in 2003)	Approximately 3,026 participants
**Framingham OA Study [54]**	Part of the Framingham Heart Study focuses on the epidemiology of OA.	Clinical assessments, imaging data	Established link between obesity and knee OA; identified genetic predispositions and lifestyle factors [65].	over 30 years (started in the 1990s)	Varies
**Chingford General Population Survey [55]**	A general population survey that includes data on OA.	Clinical assessments, imaging data	Examined the natural history and risk factors of OA and highlighted the role of estrogen and menopause in OA [239].	over 30 years (started in 1989)	Approximately 1,000 women
**ERGO [56]**	A prospective cohort study focuses on chronic disease determinants, including OA.	Clinical assessments, imaging data, biomarkers	Associations exist between OA and metabolic syndrome, cardiovascular diseases, and genetic markers [56].	3-5 years(started in 1990)	Approximately 15,000 participants
**CHECK Study [57]**	A 10-year prospective cohort study following patients with early symptomatic hip and knee OA.	Clinical assessments, radiological data, biological data	Longitudinal data on early OA identified early symptoms and risk factors for progression [66].	10 years (started in 2002)	1,002 participants
**IMI-APPROACH [58]**	A cohort study following participants to collect data on knee OA aimed at predicting OA phenotypes and progression.	Clinical assessment, Imaging, Biochemical Markers	Identifies novel OA phenotypes, validates prediction models for OA progression, and develops sensitive markers for OA progression [67, 68].	2 years (started in 2018)	297 participants
**Johnston County OA Project [59]**	A population-based study focused on OA incidence, prevalence, and progression in African-American and Caucasian residents of Johnston County, North Carolina.	Clinical assessments, X-rays, MRI, biomarkers	Identified racial and ethnic disparities in OA prevalence and progression; explored environmental factors [69, 70].	Ongoing since 1990	Approximately 3,200 participants
**Hertfordshire Cohort Study [60, 61]**	A study focusing on the health and aging of men and women born in Hertfordshire, UK, including OA.	Clinical assessments, imaging data, genetic data	Linked early life factors with OA in later life; highlighted the role of nutrition and physical activity [60, 71].	Ongoing since 1998	Approximately 3,000 participants
**TASOAC [62]**	A population-based study of older adults in Tasmania, Australia, focusing on musculoskeletal health, including OA.	Clinical assessments, imaging data, biomarkers	Assessed genetic and environmental risk factors; found strong links with bone health and physical activity [72, 73].	10.7 years (started in 2002)	1,099 participants
**NEO Study [63]**	A study focusing on the health effects of obesity, including OA, among adults in the Netherlands.	Clinical assessments, imaging data, biomarkers	Studied the impact of obesity on OA and linked adiposity measures with joint damage and pain [74].	4 years (started in 2008)	6,671 participants

**CHECK**: Cohort hip and cohort knee study; **ERGO**: Rotterdam study; **IMI-APPROACH**: Applied public-private research enabling OA clinical headway funded by the innovative; **MOST**: Multicenter Osteoarthritis Study; **MRI**: Magnetic resonance imaging; **NEO**: Netherlands epidemiology of obesity study; **OAI**: Osteoarthritis initiative; **OA**: Osteoarthritis; **TASOAC**: Tasmanian older adult cohort study; **X-rays**: X-radiation.

### 2.2 OA Changes from Spatial Perspective

#### 2.2.1 Clinical Imaging

Radiographic and clinical imaging techniques are indispensable in understanding the spatiotemporal dynamics of OA progression [76]. Traditional X-rays remain fundamental, offering valuable insights into joint space narrowing, subchondral bone sclerosis, and osteophyte formation, all hallmark features of OA. In contrast, advanced imaging modalities like MRI provide detailed visualisation of bone and soft tissue changes, enabling the early detection of cartilage degradation and synovitis, often preceding overt clinical symptoms [77, 78]. These techniques capture OA's spatial variability and temporal progression, as evidenced by longitudinal imaging studies and clinical assessments [75, 79]. For instance, radiographic assessments have been instrumental in monitoring disease progression, while MRI [77, 78], ultrasound [79], nuclear medicine [77], and arthroscopy each offer unique perspectives on OA progression.

One notable study utilising the MOST database evaluated 3,026 individuals, revealing that even in "end-stage" OA (Kellgren-Lawrence grade 4), MRI detected ongoing cartilage loss, fluctuating bone marrow lesions, effusion, and synovitis over 30 months [80]. Such findings underscore the temporal utility of imaging in tracking the evolving pathology within the joint environment. Advances in machine learning have further enhanced imaging applications; for example, a classifier achieved 78% accuracy in predicting symptomatic OA progression three years before symptoms emerged [51]. Biochemical patterns of cartilage fissuring, identified through MRI, provide additional predictive markers, enriching the clinical understanding of disease onset and progression. Spatially, imaging tools delineate affected from unaffected areas within a joint, offering a comprehensive disease map.

#### 2.2.2 Radiomics

Developing state-of-the-art techniques has propelled radiomics to the forefront of medical image analysis in OA research. Radiomics involves the high-throughput extraction of quantitative imaging features to assist in diagnosis, prognosis, and treatment planning [81, 82]. A recent study introduced and validated the Joint Space Radiology Model (JS-RM) for predicting knee OA incidence. This model integrates radiological features of the meniscus and femorotibial cartilage derived from MRI and demonstrated high predictive accuracy, achieving an area under receiver operating characteristic curve (AUC) of 0.931, sensitivity of 84.4%, and specificity of 85.6% in a test cohort [83]. Additionally, the model improved the diagnostic performance of resident doctors in interpreting MRI scans, showcasing its potential as a clinical tool. Another recent study utilised radiomics analysis of patellofemoral joint imaging data from the MOST cohort, demonstrating that integrating radiomics features significantly improved knee replacement risk prediction models (AUC increased from 0.74 to 0.81), highlighting the potential of advanced imaging techniques for personalised OA management. Other radiomics studies leveraging X-rays [84-87], Computed Tomography (CT) [88, 89], and MRI [90-92] have focused on disease classification [84-86, 89, 90], diagnosis [87, 88, 91], and progression prediction [91, 92], further illustrating the versatility of this approach.

**Figure 2.**
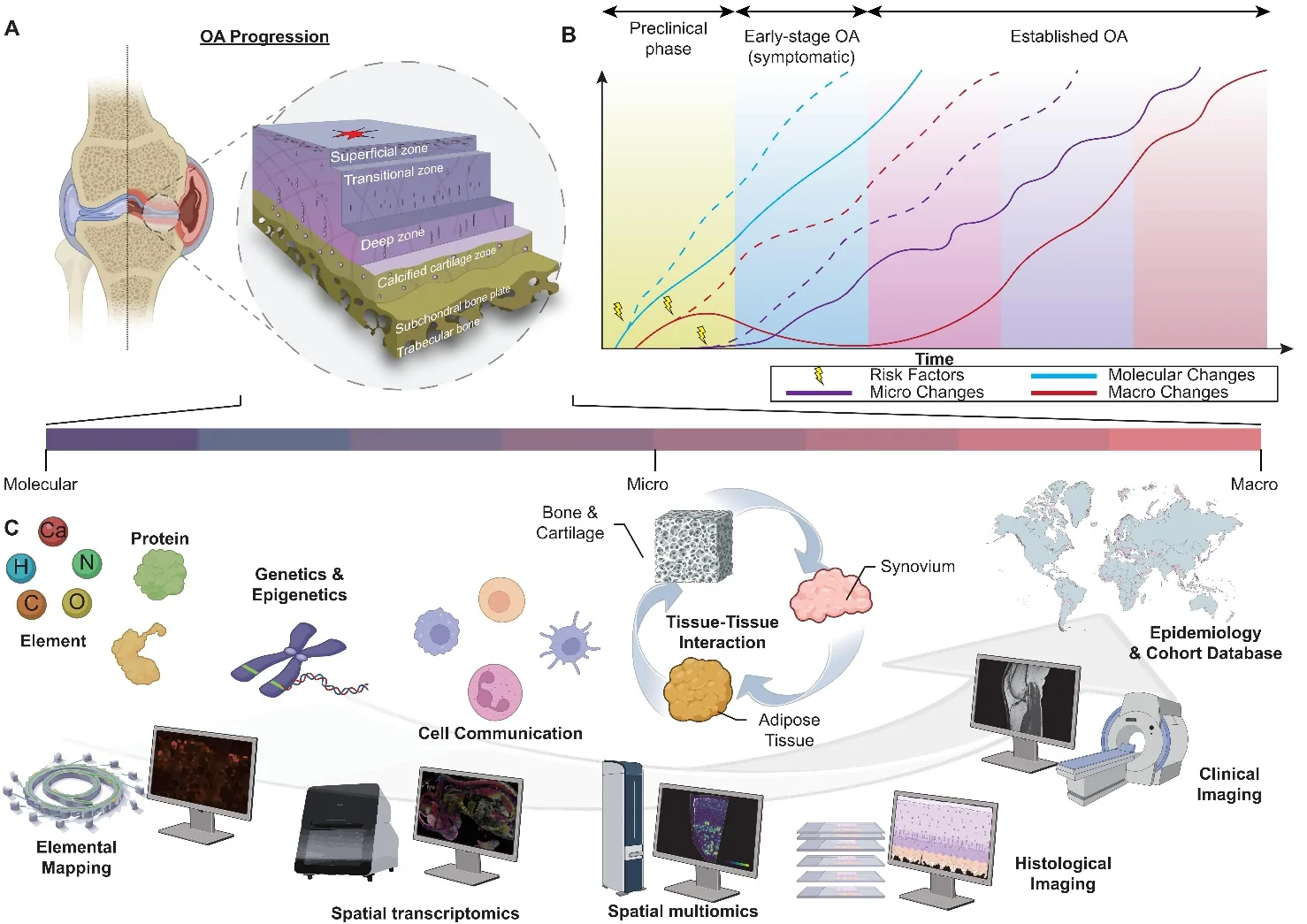
**Macro, Micro to Molecular level - Advances in understanding spatiotemporal changes of OA**. (**A**) The osteochondral unit is organised differently at the spatial level, and each layer reacts differently during OA progression, emphasising the importance of understanding spatial changes of OA. (**B**) The temporal OA progression from the preclinical phase through early-stage symptomatic OA to established OA. The risk factors (dotted/solid line) stimulate and enhance the OA progression at the molecular (blue line), micro (dotted purple line), and macro (red line) levels. The long-term temporal progression calls for a deep understanding of the disease at the temporal level. (**C**) The Multiscale Analysis approach is used for the spatiotemporal analysis of OA research. The detection scale ranges from elements, proteins and genetic/epigenetic factors to cell communication and tissue-tissue interactions. Various advanced spatially resolved techniques, such as elemental mapping, spatial transcriptomics, spatial multi-omics, histological imaging, clinical imaging, and epidemiology & cohort databases, are provided to investigate and cluster OA phenotypes and endotypes at the macro, micro and molecular levels. The figure was created with Biorender.com

#### 2.2.3 Functional Imaging

Functional imaging techniques are also gaining traction in OA research. Functional MRI [93], optical coherence tomography (OCT) [94-96], positron emission tomography-magnetic resonance imaging (PET-MRI) [97], and single-photon emission computed tomography (SPECT) [98] hold promise for dynamically tracing metabolic and inflammatory changes in OA. These techniques provide insights into the functional aspects of joint pathology, complementing structural assessments. However, additional research, standardisation of imaging feature definitions, and expanded databases are required to fully unlock their potential in early OA detection and clinical application [99].

### 2.3 Spatiotemporal Heterogeneity Provides the Basis for OA Classification

OA is a highly complex and heterogeneous disease, exhibiting significant variability across spatial and temporal dimensions. Multiple prognostic risk factors contribute to its progression, including education level, physical activity, pain severity, and bone edema observed through MRI [75, 79, 100]. Beyond these localised factors, OA’s interplay with systemic comorbidities has led some researchers to advocate for renaming it “systemic OA,” emphasising its broader systemic context and multifactorial etiology [101]. This complexity is visually represented in Figure 2, which illustrates the interconnected nature of these factors.

The classification of OA into phenotypes has been an area of active exploration, though a universally accepted standard still needs to be discovered. Several foundational studies have laid the groundwork for such classifications, combining clinical imaging, systematic reviews, and expert consensus [9, 102, 103]. These efforts have divided OA into subtypes based on medical history, pathophysiological features, and imaging findings [104-108]. However, as our understanding of OA’s etiology deepens, research increasingly focuses on integrating cross-scale data and addressing endotype (molecular or cellular features) and phenotype (observable traits) [109-115]. Table 2 provides a detailed overview of these emerging insights.

**Table 2:** Comparison of OA Subgroups Across Different Studies.

Study	Year	Number of Subgroups	Characteristics Defining Subgroups	Data Source	Sample Size	Biomarkers/Clinical Features Used	Key Findings/Unique Features	Methodology
**Hannani et al. [115]**	2024	3	Molecular endotypes based on biomarker profiles	Clinical cohort	295	Serum biomarkers, clinical assessments	Identified stable molecular endotypes in knee OA patients over time, suggesting potential for personalised treatment strategies	Longitudinal analysis of biomarker data and clinical assessments
**Nielsen et al. [49]**	2024	14	Multi-modal patient data	UK Biobank	38,372	Clinical, lifestyle, and biomarker data (GDF5, TGF-β, CRTAC1, COL9A1, etc)	Developed predictive biomarkers for OA subgroups using machine learning	Integration of diverse patient data; machine learning analysis
**Calvet et al. [109]**	2024	4	Inflammatory phenotypes	Clinical cohort	168	Cytokines in plasma and synovial fluid	Identified phenotype-specific cytokine associations with KOA severity and progression	Phenotyping based on inflammatory profiles; cytokine measurement in biological samples
**Xue et al. [110]**	2023	3	Gene expression profiles in cartilage, synovium, subchondral bone, and meniscus	GEO database	92	Gene ontology and pathway enrichment analysis	Identified three OA subtypes: bone remodelling, immune metabolism, and cartilage degradation	Unsupervised clustering; immune cell infiltration analysis; validation with qRT-PCR
**Petersen et al. [104]**	2022	4	Structural phenotypes	Clinical cohort	66	Imaging features	Proposed five structural phenotypes with different clinical implications	Imaging analysis
**Angelini et al. [67]**	2022	3	Gene expression profiles	IMI-APPROACH	297	RNA sequencing and clustering	Identified two major OA subgroups with distinct gene expression patterns	Gene expression analysis
**Knoop et al. [105]**	2022	3	Muscle strength and obesity	Clinical cohort	15	Muscle strength assessments, BMI	Explored experiences with subgroup-specific exercise therapy	Qualitative interviews with patients and clinicians
**Cao et al. [111]**	2022	3	Synovial lipid metabolism disorder and fibroblast-like synoviocyte dysfunction	GEO database	70	Synovial fluid analysis, gene expression profiling	Identified a specific OA subtype with distinct synovial lipid metabolism and synoviocyte dysfunction	Synovial fluid analysis, gene expression profiling
**Werdyani et al. [112]**	2021	3	Plasma metabolomics	Clinical trials	852	Metabolomic analysis of plasma samples	Defined endotypes of primary OA based on metabolomic profiles	Metabolomic analysis
**Vongsirinavarat et al. [106]**	2020	4	Levels of activity limitation	Clinical cohort	250	Pain intensity, range of motion, muscle strength	Classified individuals into four phenotypes based on disability levels	Cluster analysis of activity limitations; comparison of impairments and participation restrictions
**Yuan et al. [113]**	2020	4	Transcriptomic profiles of knee joint tissues	Tissue samples	106	Gene expression analysis	Identified four OA subtypes: inflammatory, cartilage metabolic, chondrocyte differentiation, and Wnt signalling	Integrated transcriptomic analysis of cartilage, synovium, and subchondral bone tissues
**Radojčić et al. [107]**	2020	4	Pain progression patterns	VIDEO trial and OAI database	5270	Longitudinal pain assessment	Defined OA subgroups based on distinct pain trajectories	Longitudinal study
**Soul et al. [114]**	2018	2	Gene expression profiles	Articular cartilage samples	44	Genome-wide expression analysis	Discovered two distinct OA subgroups with differing pathogenic pathways	Unsupervised clustering of RNA-Seq data; pathway analysis
**Dell’Isola et al. [116]**	2016	6	Chronic pain, inflammation, metabolic syndrome, bone and cartilage metabolism, mechanical overload, minimal joint disease	Systematic review	NA	Clinical variables, biomarkers, imaging findings	Identified six distinct clinical phenotypes in knee OA	Systematic review of existing literature
**Wyatt et al. [108]**	2016	4	Histopathological subgroups	Post-mortem donation and patient recruitment	486	Tissue analysis of knee joints	Identified subgroups based on distinct histopathological characteristics	Tissue analysis

**BMI**: Body mass index; **COL9A1**: Collagen type IX alpha 1 chain; **CRTAC1**: Cartilage acidic protein 1; **GDF5**: Growth differentiation factor 5; **GEO**: Gene Expression Omnibus; **IMI-APPROACH**: Applied Public-Private Research Enabling **OA** Clinical Headway funded by the Innovative; **NA**: Not applicable; **OA**: Osteoarthritis; **OAI**: Osteoarthritis Initiative; **qRT-PCR**: Quantitative reverse transcription polymerase chain reaction; **RNA**: Ribonucleic acid; **TGF-β**: Transforming growth factor beta; **VIDEO**: The Vitamin D Effect on Osteoarthritis.

One noteworthy example is applying the Subtype and Stage Inference (SuStaIn) model to OA classification [9]. Using data from the OA Initiative (OAI) database, this machine learning technique employed X-rays and MRI imaging to identify three distinct knee OA subtypes. These subtypes include early pain (15% of cases), structural damage with concurrent pain (61%), and late pain (24%). Each subtype reflects unique clinical symptoms and structural damage patterns at different stages of disease progression. This classification approach underscores the potential of advanced computational models to unravel the complexity of OA heterogeneity. Beyond the SuStaIn model, a systematic review has proposed additional phenotype categorisations based on OA’s leading risk factors [116]. These include six major phenotypes: (1) chronic pain dominated by central mechanisms like central sensitisation, (2) inflammatory phenotypes characterised by elevated inflammatory biomarkers, (3) metabolic syndrome-associated phenotypes linked to obesity, diabetes, and metabolic disturbances, (4) local bone and cartilage metabolic alterations, (5) mechanical overload-driven phenotypes predominantly associated with varus malalignment and medial compartment disease, and (6) minimal joint disease phenotypes marked by mild symptoms and slow progression over time. Similarly, Ali et al. have proposed an alternate phenotypic framework emphasising pathophysiological drivers. Their classification includes six subtypes: (1) aging-driven phenotype, (2) cartilage-driven phenotype, (3) metabolic phenotype, (4) synovitis-driven inflammatory phenotype, (5) traumatic injury-driven phenotype, and (6) subchondral bone phenotype [117, 118]. This categorisation highlights the diverse mechanisms underlying OA progression and aligns with growing interest in personalised medicine. A recent addition to OA classifications is the accelerated knee OA subtype. This rapidly progressing form of OA is characterised by a transition from no radiographic evidence to advanced disease within four years [119]. Researchers have contextualised this subtype by comparing it to typical, slower-progressing OA cases and exploring potential factors such as aging, previous case reports, and relevant animal models. Despite these advancements, the field still lacks widely recognised and validated subcategories. Achieving this requires a multidisciplinary effort, integrating insights from diverse fields such as molecular biology, imaging, epidemiology, and clinical practice. A consensus-based approach, supported by input from field experts and robust validation, is essential for defining precise subcategories. This is critical for enhancing early diagnosis and accurate disease management and reducing the overall burden of OA on patients and healthcare systems.

### 2.4 Challenges in Integrating Cohort Data and Radiomics in OA

While promising, the above imaging strategies face several limitations that hinder their widespread adoption and effectiveness. A key issue is the lack of standardisation across feature extraction, data processing, and model evaluation, which complicates comparisons between studies and institutions. Additionally, the availability of high-quality, annotated datasets is limited, with small sample sizes and insufficient diversity reducing generalizability. Variability in imaging protocols, equipment, and settings between institutions introduces inconsistencies that challenge validation and clinical implementation. Computational complexity further adds to these challenges, as advanced models demand significant resources and expertise that are not universally accessible. Despite advancements, clinical integration still needs to be improved. Radiomics models often lack real-world validation and seamless incorporation into workflows, leading to hesitation among clinicians, mainly due to the "black box" nature of many machine learning models. Overfitting and reproducibility issues persist, especially in studies with small datasets, emphasising the need for more extensive and varied cohorts to ensure robust findings. Advanced imaging techniques, such as PET-MRI or OCT, come with their own set of challenges. These include high costs, specialised equipment requirements, and limited accessibility in routine healthcare settings. Furthermore, functional imaging techniques often lack extensive longitudinal data, particularly for OA research. Furthermore, radiomics features require further validation to confirm their clinical relevance and standardised imaging databases are essential for accurate modelling and inter-institutional validation. Addressing these limitations necessitates collaborative efforts focused on standardising protocols, enhancing dataset quality, improving interpretability, and validating results across diverse clinical environments. Such advancements will be critical for transitioning radiomics and clinical imaging into routine clinical practice, fulfilling their potential in early and precise OA diagnostics.

In summary, while substantial progress has been made in understanding OA’s heterogeneity and identifying phenotypes, many challenges remain. Future efforts should prioritise refining these classifications, validating them through cross-disciplinary collaboration, and leveraging advanced methodologies to integrate endotypes and phenotypes. Such advancements will pave the way for more targeted interventions, improving outcomes for individuals with OA.

## 3. Spatiotemporal Development Trajectory in OA at The Micro Level

Over recent decades, researchers have sought to delineate the development trajectories of OA at the histopathological level by employing various grading systems. These systems primarily fall into two categories: those following sequential stages of increasing OA severity [120, 121] and those aggregating independent indicators of OA severity [122, 123]. The Modified Mankin score, and the Osteoarthritis Research Society International (OARSI) grading system are the most renowned. Scientists have successfully built various OA grading systems in humans [121], horses [124], dogs [125], rabbits [126] and rats [127], and the grading systems have covered most of the tissues and cell types changed within the joint. The elements involved in the changes include cartilage, tidemark, proteoglycan, collagen integrity, subchondral bone plate, meniscus, synovium, and osteophytes, which can be seen via histopathological staining.

### 3.1 Cartilage

Normal: At the histopathological level, cartilage is the core tissue affected during OA progression. It is a highly differentiated tissue that maintains a high regeneration capacity during development and architecture after birth but loses its regenerative capacity during OA progression [128-130]. Therefore, genetic and transcriptomic pivots during development and growth are crucial to understanding OA changes [131-133]. Chondrogenesis is a well-coordinated process essential for embryonic development, adult cartilage maintenance, and vertebrate repair. Chondrocytes' fate determination and differentiation involve the differential expression of genes crucial at each stage of chondrogenesis [134]. During chondrogenesis, SRY-Box Transcription Factor 9 (SOX9) was believed to be crucial in endochondral ossification and bone elongation [135], owing to its key function in the initiation of chondrocyte differentiation [136], activation of cartilage matrix genes [137, 138], maintenance of chondrocyte phenotype [136], inhibition of hypertrophy and osteogenic differentiation [138, 139] and interaction with other factors [137, 138]. A recent in situ study showed that SRY-Box Transcription Factor 8 (SOX8) is also expressed at levels comparable to SOX9 in reserve and early columnar chondrocytes [140]. The study showed that SOX8 expression stops as SOX9 peaks in late columnar and hypertrophic chondrocytes, and both SOX8 and SOX9 are essential for skeletal growth by promoting the conversion of growth plate chondrocytes to actively proliferating columnar cells [140].

Modern Technology, including single-cell RNA-sequencing (scRNA-seq) [141] and spatial transcriptomics [142-144], has improved our understanding of cartilage structure and its cell subpopulation at the spatial level. The distribution of chondrocyte subpopulations in normal cartilage reveals distinct regional preferences. Prehypertrophic chondrocytes (preHTC), inflammatory chondrocytes (InfC), pre-inflammatory chondrocytes (preInfC), and fibrocartilage chondrocytes (FC) predominantly occupy the surface and superficial layers [144]. In contrast, proliferative chondrocytes (ProC), hypertrophic chondrocytes (HTC), regulatory chondrocytes (RegC), and pre-inflammatory chondrocytes (preInfC) are significantly more abundant in these regions compared to the middle and deep layers. Spatial transcriptomics shows that preHTC, InfC, preInfC, and FC cells are abundant in weight-bearing (WB) and non-weight-bearing areas but less in normal controls. Hypertrophic chondrocytes (HTC), enriched with genes related to extracellular matrix organisation and ossification, are significantly more prevalent in the deep cartilage region. Similarly, RegC cells, associated with degradation processes such as amino sugar metabolism, are also more abundant in the deep area.

OA: In the early stage of OA, cartilage retains its surface integrity but may exhibit superficial fibrillation, such as microscopic cracks [121]. This phase also shows signs of matrix edema, leading to alterations in matrix density and staining properties with cationic dyes, reflecting early proteoglycan changes. Chondrocyte proliferation and apoptosis may be observed, particularly in superficial layers [145, 146]. Progression to the next stage involves the focal loss of the superficial matrix, evidenced by the spallation of small matrix fragments and the beginning of surface layer discontinuity. This stage may also display deep fibrillation extending through the superficial zone, with matrix staining changes indicating altered biochemical composition [145]. As OA advances, fissures extend into the mid zone, forming vertical clefts that may branch. This results in more pronounced heterogeneity in matrix composition adjacent to fissures, with areas of proteoglycan depletion and increased staining. Chondrocyte death and complex chondron formations become more apparent [147, 148]. These processes are often accompanied by increased chondrocyte death and matrix degradation. The most advanced stage is characterised by total loss of hyaline cartilage, exposing the underlying bone, which may exhibit increased density and thickness [121]. This stage may also involve fibrocartilage repair, which can fill the eroded cartilage volume. In the final stage, significant deformation of the joint surface occurs due to continued bone microfractures and reparative changes, including the formation of osteophytes. These osteophytes and fibrocartilaginous proliferation contribute to altering joint contour and function.

**Table 3:** Cartilage single-cell changes at the spatiotemporal level in OA progression.

Tissue	Cell Type / Subpopulation	Location	Markers	Function	Early OA	Advanced OA
Chondrocyte [141, 144]	ProCs	AS	BMP2, HMGA1, FOSL1	Differentiation, repair;	Increased in SZ; repair signals (BMP2 elevated); markers similar to normal	Declines; proliferative potential reduced
	**ECCs**	MZ	FRZB, CHRDL2	Cartilage development; Markers	Slight increase in MZ; enhanced cartilage matrix synthesis	Maintains in MZ; no significant functional shift
	**HomCs**	DZ	HSPA1B, HSPA6, JUN	Protein folding, homeostasis;	Decreases in DZ; reduced homeostasis	Absent in DZ; loss of maintenance capability
	**RegCs**	SZ	CHI3L1, CHI3L2	Catabolism modulation	Decreases in SZ; lower catabolic activity	Minimal presence; catabolism shifted to other cell types
	**RepCs**	Across zones	CILP, OGN	Repair after damage	Mild increase in SZ and MZ; response to initial damage	Reduced in MZ; overwhelmed by matrix degradation
	**preHTCs**	DZ	PRG4, ABI3BP, CRTAC1	Cartilage mineralization precursor	Shifts to AS; early ossification signals (TGF-β pathway active)	Strong increase in AS and MZ; promotes hypertrophic differentiation
	**HTCs**	DZ	IBSP, COL10A1	Ossification	Found in DZ; mild activation of ossification markers	Overabundant in DZ and AS; drives matrix degradation and calcification
	**FCs**	DZ	COL1A1, COL1A2	Angiogenesis regulation	Emerges in AS and MZ; increased fibrocartilage markers	Dominates MZ and DZ; increases matrix stiffness and abnormal repair
	**preFCs**	DZ	COL27A1, WWP2	ECM precursor	Higher in AS; contributes to early ECM remodelling	Declines; replaced by hypertrophic or inflammatory chondrocytes
	**preInfCs**	Absent in normal cartilage	IFI16, IFI27	NA	Emerges in AS and SZ; early inflammation markers (IFI27 linked to apoptosis)	Found in MZ; transitions into inflammatory chondrocytes
	**InfCs**	Absent in normal cartilage	CXCL8, CD74, GPR183	NA	Absent; early precursor stage seen as preInfC	Dominates in MZ; contributes to inflammatory cascades, MIF-CD74 signalling

**ABI3BP**: ABI family member 3 binding protein; **AS**: Articular surface; **BMP2**: Bone morphogenetic protein 2; **CD74**:Cluster of differentiation 74; **CHI3L1**: Chitinase 3 like 1; **CHI3L2**: Chitinase 3 like 2; **CILP**: Cartilage intermediate layer protein; **COL1A1**: Collagen type I alpha 1 chain; COL1A2: Collagen type I alpha 2 chain; **COL10A1**: Collagen type X alpha 1 chain; **COL27A1**: Collagen type XXVII alpha 1 chain; **CRTAC1**: Cartilage acidic protein 1; **DZ**: Deep zone; **ECM**: Extracellular matrix; **ECCs**: Effctor chondrocytes; **FCs**: Fibrocartilaginous chondrocytes; **FOSL1**: Fos like 1, AP-1 transcription factor subunit; **FRZB**: Frizzled related protein; **GPR183**: G protein-coupled receptor 183; **HMGA1**: High mobility group AT-hook 1; HomCs: Homeostatic chondrocytes; **HSPA1B**: Heat shock protein family A (Hsp70) member 1B; **HSPA6**: Heat shock protein family A (Hsp70) member 6; **HTCs**: Hypertrophic chondrocytes; **IBSP**: Integrin binding sialoprotein; **IFI16**: Interferon gamma inducible protein 16; **IFI27**: Interferon alpha inducible protein 27; **InfCs**: Inflammatory chondrocytes; **JUN**: Jun proto-oncogene, AP-1 transcription factor subunit; **MIF**: Macrophage migration inhibitory factor; **MZ**: Middle zone; **NA**: Not applicable; **OGN**: Osteoglycin; **preFCs**: Prefibrocartilaginous chondrocytes; **preHTCs**: Prehypertrophic chondrocytes; **preInfCs**: Pre-inflammatory chondrocytes; **PRG4**: Proteoglycan 4; ProCs: Proliferative chondrocytes; **RegCs**: Regulatory chondrocytes; **RepCs**: Reparative chondrocytes; **SZ**: Superficial zone; **WWP2**: Ww domain containing E3 ubiquitin protein ligase 2.

State-of-the-art techniques, including scRNA-seq [141] and multi-omics data integration [144], provided further evidence of chondrocyte cell population changes (Table 3). In early OA, proliferative chondrocytes (ProCs) increase in the superficial zone as part of an initial reparative response. However, as OA progresses, the population of ProCs declines, resulting in reduced proliferative potential. Effector chondrocytes (ECCs), involved in cartilage matrix synthesis, exhibit a modest increase in the middle zone during early OA but remain relatively stable in advanced OA without significant functional changes. Homeostatic chondrocytes (HomCs), critical for maintaining protein folding and overall homeostasis, decrease in the deep zone during early OA and become absent in advanced OA, reflecting a loss of homeostatic capacity. Regulatory chondrocytes (RegCs), responsible for modulating catabolic processes, also decrease in the superficial zone during early OA and are minimally present in advanced OA as other cell types take over catabolic functions. Reparative chondrocytes (RepCs), which contribute to extracellular matrix (ECM) repair, show a mild increase in the SZ and MZ during early OA in response to initial cartilage damage. However, in advanced OA, RepCs are significantly reduced as they become overwhelmed by extensive matrix degradation. PreHTCs shift from the deep layer to the joint surface in the early stages of OA, exhibiting early ossification signals mediated by activation of TGF-β pathways. In advanced OA, preHTCs markedly increase in the articular surface and middle zone, promoting hypertrophic differentiation. HTCs, involved in ossification, show mild activation in the deep zone during early OA but become overabundant in both the deep zone and articular surface as OA progresses, contributing to significant matrix degradation and calcification. Fibrocartilage chondrocytes (FCs) emerge in the articular surface and middle zone in early OA, contributing to increased fibrocartilage-specific markers and eventually dominating the middle zone and deep zone in advanced OA, resulting in increased matrix stiffness and maladaptive repair processes. Prefibrocartilage chondrocytes (preFCs), which play a role in early ECM remodelling, are more prevalent in the AS during early OA but decline in advanced OA as hypertrophic or inflammatory chondrocytes replace them. Pre-inflammatory chondrocytes (preInfCs), absent in normal cartilage, emerge in the articular surface and superficial zone during early OA and are linked to apoptosis via IFI27 expression. In advanced OA, preInfCs differentiate into inflammatory chondrocytes (InfCs), which dominate the middle zone, contributing to inflammatory cascades, including macrophage migration inhibitory factor (MIF)-cluster of differentiation 74 (CD74) signalling [144].

### 3.2 Synovium

**Normal**: Under physiological conditions, the synovium consists primarily of two major synoviocyte types: Type A and Type B synoviocytes [149]. Type A synoviocytes, also called macrophage-like synoviocytes, are mainly involved in phagocytosis and debris clearance. In contrast, Type B synoviocytes, which are fibroblast-like, are responsible for synthesising synovial fluid and maintaining extracellular matrix (ECM) components, including collagen. These synoviocytes maintain synovial homeostasis, providing an optimally lubricated environment crucial for joint function.

**OA**: In OA progression, the synovium undergoes significant spatiotemporal changes, encompassing alterations in cellular subpopulations and their functional dynamics (Table 4). As OA progresses, multiple macrophage subpopulations emerge within the synovium. In early OA, Transitional Macrophages (T-Mϕ) are important in initiating inflammatory responses. These macrophages express markers with both pro-inflammatory and anti-inflammatory properties, highlighting their transitional nature. In advanced OA, T-Mϕ exhibits increased activity, contributing to persistent and chronic inflammation. Another subpopulation, Fibrotic Immune-Regulated Macrophages (IR-Mϕ), is implicated in tissue repair and immune regulation. Their presence is limited in early OA but becomes more prominent in advanced stages, significantly contributing to synovial fibrosis. Interferon-stimulated macrophages (IFNS-Mϕ), are moderately abundant in early OA and increase in presence as OA progresses, exacerbating inflammatory responses. S100A8/9hi Macrophages (S100A8/9hi-Mϕ) are involved in auto debridement, facilitating the clearance of damaged tissue. Their levels increase significantly in advanced OA, corresponding with ongoing tissue damage and remodelling. Type B synoviocytes are localised in the synovial lining layer. During early OA, synovial fibroblasts activate, proliferate, and secrete ECM proteins that facilitate tissue repair. They also mediate inflammation by secreting pro-inflammatory cytokines. In advanced OA, fibroblast activity is marked by increased production of fibrotic ECM, leading to synovial fibrosis. These fibroblasts also contribute to chronic inflammation through enhanced secretion of inflammatory cytokines, such as IL-6 and Interleukin 8 (IL-8), which promote synovial hyperplasia and joint stiffness. Cluster of Differentiation 4 (CD4)+ T cells are key modulators of immune responses and differentiate into various helper subtypes, including Type 1 T-helper cell (Th1), Type 2 T-helper cell (Th2), Type 17 T-helper cell (Th17), and regulatory T cells (Tregs). In early OA, naive and memory CD4+ T cells are predominant. In contrast, in advanced OA, increased activation occurs, with regulatory subtypes (e.g., Tregs) playing a more significant role in immune regulation. CD8+ T cells also exhibit increased expression of cytotoxic and proliferative markers during early OA. They are predominantly found as effector memory T cells in advanced OA, suggesting an ongoing immune response. In early OA, B cells are present at relatively low levels, indicating a minimal contribution to the early inflammatory milieu—however, their abundance increases in advanced OA, suggesting an enhanced role in sustaining chronic inflammation. In summary, the synovium undergoes dynamic cellular alterations as OA progresses, with different synoviocyte populations contributing to inflammation, fibrosis, and tissue remodelling.

**Table 4:** Synovium single-cell changes at the spatiotemporal level in OA progression.

Tissue	Cell Type / Subpopulation		Location	Markers	Function	Early OA	Advanced OA
Synovium [240]	Type A Synoviocytes (Macrophage-like)	Transitional Macrophages (T-Mφ)	NA	CCL3, CCL3L1, IGF1, MRC1	Display both pro-inflammatory and anti-inflammatory markers, indicating a transitional role	Present, playing a role in early inflammation	Increased activity contributing to chronic inflammation
		Fibrotic fibrotic immune-regulated macrophages (IR-Mφ)	NA	FN1, SPP1	Involved in tissue repair and immune regulation	Limited presence, initiating fibrotic changes	Prominent, driving fibrosis in synovial tissue
		Interferon-stimulated macrophages (IFNS-Mφ)	NA	EPSTI1, STAT1, MX1, IFI44L, ISG15	Involved in inflammatory responses	Present at moderate levels	Increased presence, contributing to inflammation
		S100A8/9hi macrophages (S100A8/9hi-Mφ)	NA	SERPINB2, CD52, S100A8, S100A9	Involved in auto debridement, indicating a "clean up" function	Present at low levels	Elevated levels associated with tissue damage and auto debridement
	Type B Synoviocytes (Fibroblast-like)	Fibroblast	Synovial Lining layer	PDPN, THY1, COL1A1, COL3A1, FAP	Production of extracellular matrix (ECM) components (e.g., collagen), Maintenance of synovial fluid homeostasis, Mediation of fibrosis and inflammation in response to cytokines	- Activated fibroblasts secrete ECM proteins for tissue repair - Proliferation and early inflammatory response are noted	- Increased production of fibrotic ECM leading to synovial fibrosis - Enhanced inflammatory cytokine secretion (e.g., IL-6, IL-8) promoting chronic inflammation - Contribution to synovial hyperplasia and joint stiffness
	T Cells	CD4+ T Cells	NA	CD4, GATA3, IL4R, FOXP3, IL21R, TBX21	Differentiates into various helper subtypes (Th1, Th2, Th17, Treg), regulates immune responses	Higher proportion of naïve and memory CD4+ T cells; differentiation-related markers (e.g., IL-2, IL17A)	Increased presence of activated and regulatory subtypes (e.g., Tregs) involved in immune regulation
		CD8+ T Cells	NA	CD8, GZMB, NKG7, CCL5, IL7R, TCF7	Cytotoxic activity, effector functions in response to antigens	Higher expression of cytotoxic and proliferating markers	Predominantly effector memory T cells (TEM), indicating a long-term immune presence
	B Cells		NA	CD19, CD20, CD79A, CD27	Antibody production, immune response modulation	Limited presence, suggesting a minimal contribution to inflammation	Increased presence, suggesting involvement in chronic inflammation

**B Cells**: B lymphocytes; **CCL3**: C-C motif chemokine ligand 3; **CCL3L1**: C-C motif chemokine ligand 3 like 1; **CD4**: Cluster of differentiation 4; **CD8**: Cluster of differentiation 8; **CD19**: Cluster of differentiation 19; **CD20**: Cluster of differentiation 20; **CD27**: Cluster of differentiation 27; **CD52**: Cluster of differentiation 52; **CD79A**: Cluster of differentiation 79A; **COL1A1COL1A1**: Collagen type I alpha 1 chain; **COL3A1**: Collagen type III alpha 1 chain; **EPSTI1**: Epithelial stromal interaction 1; **FAP**: Fibroblast activation protein alpha; **FN1**: Fibronectin 1; **FOXP3**: Forkhead box P3; **GATA3**: GATA binding protein 3; **GZMB**: Granzyme B; **IFI44L**: Interferon-induced protein 44-like; **IGF1**: Insulin-like growth factor 1; IL-2: Interleukin 2; **IL-6**: Interleukin 6; **IL-8**: Interleukin 8; **IL-17A**: Interleukin 17A; **IL21R**: Interleukin 21 receptor; **IL4R**: Interleukin 4 receptor; **IR-Mφ**: Immune-regulated macrophages; **ISG15**: Interferon-stimulated gene 15; **MRC1**: Mannose receptor C-type 1; **MX1**: Myxovirus resistance protein 1; **NA**: Not applicable; **NKG7**: Natural killer cell group 7 sequence; **PDPN**: Podoplanin; **S100A8**: S100 calcium-binding protein A8; **S100A9**: S100 calcium-binding protein A9; **SERPINB2**: Serpin family B member 2; **SPP1**: Secreted phosphoprotein 1 (osteopontin); **STAT1**: Signal transducer and activator of transcription 1; **TBX21**: T-box transcription factor 21; Th: T-helper cell; **T-Mφ**: Transitional macrophages; Tregs: Regulatory T cells; **THY1**: Thy-1 cell surface antigen; **TCF7**: Transcription factor 7.

### 3.3 Subchondral Bone Plate

**Normal**: Under normal physiological conditions, subchondral bone exhibits a low turnover rate, which is essential for maintaining a stable microenvironment and supporting overall joint function. Osteocytes, residing within the bone matrix, are necessary for bone maintenance and mechanosensation. Regulating bone remodelling through signalling pathways involves markers such as sclerostin (SOST) and dentin matrix protein 1 (DMP1). The cellular constituents of subchondral bone, including osteoblasts (OBs), osteoclasts (OCs), mesenchymal stem cells (MSCs), endothelial cells (ECs), and immune cells, are typically maintained in a homeostatic state with minimal pathological activity [150].

The subpopulations of cells within the subchondral bone reside in specific niches, reflecting their specialised roles. Endothelial cells are located in the vascular regions of the subchondral bone, with two subpopulations identified: kinase insert domain receptor (KDR)-negative cells, which are associated with inflammation, and KDR-positive cells, which are involved in angiogenesis. Osteoblasts are present in different regions depending on their specific subtype. Endothelial osteoblasts (EnOBs) are found near blood vessels and contribute to vascularisation, stromal osteoblasts (StOBs) are located in areas of extracellular matrix production, and mineralising osteoblasts (MinOBs) are found in regions of active mineral deposition. Mesenchymal stem cells are typically located near areas of bone damage and early lesions, where they differentiate into osteoblasts or chondrocytes, contributing to bone formation and repair. These distinct subpopulations occupy different niches within the subchondral bone, ensuring proper bone function under normal physiological conditions.

**OA**: The progression of OA involves cellular and subpopulation changes within the subchondral bone, leading to significant alterations in bone structure and function [151] (Table 5). In the initial stages of OA, osteoblasts have mild activation, contributing to early bone thickening and subchondral sclerosis. Osteoblasts at this stage are characterised by the upregulation of markers involved in bone formation and early osteophyte development [152]. Osteoclasts exhibit increased resorptive activity in response to microdamage [153]. This resorption is necessary to remove damaged bone and facilitate subsequent remodelling. MSCs are activated in early lesions, differentiating into osteoblasts to facilitate early repair processes and promote initial bone formation. The recruitment and differentiation of MSCs are driven by markers such as melanoma cell adhesion molecule (MCAM) and CD146, which are critical in osteoblast lineage commitment [154]. Endothelial cells (ECs) exhibit early angiogenic responses around areas of bone damage, enhancing nutrient supply to the affected regions through increased expression of angiogenesis markers [155]. Immune cells, including T cells, B cells, and macrophages, are present in limited numbers, contributing to localised cytokine production that remains relatively minor at this stage. However, this early immune response sets the stage for further immune-mediated processes exacerbating disease progression. In advanced OA, osteoblast activity becomes markedly increased, leading to the formation of osteophytes and significant subchondral bone sclerosis. Osteoblasts at this stage are highly active, contributing to extensive bone formation and mineralisation, often leading to joint deformities. Osteoclast activity persists, resulting in substantial bone resorption, increased microdamage, and extensive remodelling. Persistent osteoclast activity is marked by elevated levels of catabolic enzymes, particularly cathepsin K, which is essential for bone matrix degradation and contributes to the pathological remodelling of subchondral bone. Endothelial cells undergo pronounced angiogenesis, enhancing nutrient supply that supports inflammation and advanced bone remodelling. This angiogenesis is marked by upregulated vascular endothelial growth factor (VEGF) signalling and increased formation of vascular structures, which further contributes to subchondral bone pathology. There is heightened recruitment of MSCs, which accelerates osteoblast differentiation and fosters osteophyte formation, contributing to joint deformity. MSCs are recruited and differentiated at an increased rate, driven by an altered microenvironment characterised by inflammatory signals and mechanical stress.

**Table 5:** Subchondral bone single-cell changes at the spatiotemporal level in OA progression.

Tissue	Cell Type / Subpopulation	Location	Markers	Function	Early OA	Advanced OA
**Calcified Cartilage zone [151]**	Chondroclasts	Calcified cartilage	MMP9, CTSK	Degradation of cartilage matrix	NA	Increased presence contributes to cartilage degradation
Subchondral Bone [151]	Osteocytes	Embedded within the bone matrix	SOST, DMP1	Bone maintenance, mechanosensation	NA	Altered signalling, impaired bone quality
	**OBs**	NA	RUNX2, CDH11	Bone formation	Mild osteoblast activation leads to initial bone thickening and sclerosis.	Extensive osteoblast activity with formation of osteophytes and significant subchondral bone sclerosis.
	**OCs**	NA	TRAP, cathepsin K, RANKL	Bone resorption	Mild osteoclast activity in response to early microdamage and resorption near damaged areas.	Persistent osteoclast activity results in substantial bone resorption, subchondral microdamage, and bone remodelling.
	**Bone Lining Cells**	Bone surface	SOST, RANKL	Regulation of bone formation and resorption	NA	Altered activity contributes to impaired bone homeostasis
	**MSCs**	NA	MCAM, CD146	Differentiate into osteoblasts and chondrocytes.	MSC activation near early lesions, differentiating into osteoblasts for initial repair.	High MSC recruitment, accelerating osteoblast differentiation, promoting osteophyte formation and joint deformity.
	**ECs**	NA	PECAM1, CD31, KDR.	Formation and maintenance of blood vessels.	Initial angiogenesis around damaged bone, with a slight increase in nutrient supply.	Extensive angiogenesis, supplying nutrients that support inflammation and advanced bone remodelling.
	**Immune Cells (T cells, B cells, Macrophages)**	Immune modulation	NA	NA	Limited immune cell presence with minor cytokine production in early lesions.	High immune infiltration and significant cytokine production exacerbate osteoclast-driven bone resorption and remodelling.

**B Cells**: B lymphocytes; **CD146**: Cluster of differentiation 146; **CDH11**: Cadherin 11; **CTS K**: Cathepsin K; **DMP1**: Dentin matrix acidic phosphoprotein 1; **ECs**: Endothelial cells; **KDR**: Kinase insert domain receptor; **MCAM**: Melanoma cell adhesion molecule; **MMP9**: Matrix metallopeptidase 9; **MSCs**: Mesenchymal stem cells; **NA**: Not applicable; **OBs**: Osteoblasts; **OCs**: Osteoclasts; **PECAM1**: Platelet and endothelial cell adhesion molecule 1; **RANKL**: Receptor activator of nuclear factor kappa-Β ligand; **RUNX2**: Runt-related transcription factor 2; **SOST**: Sclerostin; T Cells: T lymphocytes; **TRAP**: Tartrate-resistant acid phosphatase.

Recent studies using single-cell RNA sequencing have revealed significant changes in the subpopulations of cells within the subchondral bone during OA progression [151]. Two distinct subpopulations in the endothelial cell compartment have been identified: kinase insert domain receptor (KDR)-negative endothelial cells characterised by an inflammatory response and KDR-positive endothelial cells associated with angiogenesis. The KDR-positive endothelial cells become increasingly prominent in advanced OA, indicating aberrant vascularisation. Osteoblasts can be further divided into endothelial osteoblasts (EnOBs), stromal osteoblasts (StOBs), and mineralising osteoblasts (MinOBs). Endothelial osteoblasts are involved in vascularisation, stromal osteoblasts contribute to extracellular matrix production, and mineralising osteoblasts are critical for mineral deposition. The relative abundance of these osteoblast subpopulations changes during OA progression, with endothelial osteoblasts predominating at early stages and mineralising osteoblasts becoming more prevalent in advanced stages as bone mineralisation and sclerosis progress. Mesenchymal stem cells also exhibit increased heterogeneity, with specific subpopulations differentiating more actively into osteoblasts or chondrocytes in response to inflammatory signals and mechanical stress. These changes in subpopulations reflect a dynamic remodelling process aimed at repairing damage, leading to pathological bone changes and joint deterioration. Additionally, there is extensive infiltration of immune cells, with significant production of pro-inflammatory cytokines, including tumour necrosis factor-alpha (TNF-α) and interleukin-1 beta (IL-1β), that exacerbate osteoclast-mediated bone resorption and remodelling. These cytokines further drive the inflammatory cascade, disrupting bone homeostasis and promoting chronic inflammation. In summary, targeting the distinct cellular and subpopulation changes within the subchondral bone may offer promising avenues for therapeutic interventions to prevent or slow the progression of OA.

### 3.4 Infrapatellar Fat Pad

**Normal**: The infrapatellar fat pad (IPFP) is a unique white adipose tissue in the knee joint, below the patella and behind the patellar ligament [156]. Under physiological conditions, the IPFP serves critical biomechanical and homeostatic functions, including acting as an impact dampener, facilitating the dispersion of synovial fluid, and contributing to joint stability. It is a highly vascularised tissue comprising various cellular populations, such as adipocytes, fibroblasts, immune cells, and endothelial cells. These diverse cell types are integral to maintaining knee joint integrity by modulating inflammatory responses, facilitating tissue repair, and providing structural support. The IPFP's close anatomical and functional association with the synovium forms an integrated unit that plays a vital role in joint lubrication and movement, underscoring its significance in overall knee health.

**OA**: During OA progression, the IPFP undergoes profound cellular and molecular changes contributing to the pathogenesis of OA [156]. OA induces a profibrotic and inflammatory phenotype in the IPFP, marked by increased fibrosis, chronic low-grade inflammation, and altered cellular heterogeneity. Intermediate fibroblasts emerge as key drivers of OA-associated fibrosis, while macrophages and other immune cells exacerbate inflammation by releasing pro-inflammatory cytokines. The transcriptomic landscape of the IPFP shifts markedly, with the upregulation of genes associated with fibrosis and inflammation, including those involved in the transforming growth factor-beta (TGF-β) signalling pathway [157]. These molecular alterations, combined with increased adipokines and other inflammatory mediators, exacerbate cartilage degradation and OA progression, highlighting the IPFP's pivotal role in OA pathogenesis.

Spatiotemporal changes at the micro level in the IPFP during OA progression further elucidate the pathological transformation of this tissue [157, 158] (Table 6). In early OA, precursor-like fibroblasts are distributed throughout the IPFP and contribute to tissue repair, shock absorption, and joint support. However, these fibroblasts exhibit increased expression of fibrosis-associated markers and inflammatory activation as OA advances. In late-stage OA, these cells reach an advanced differentiation state, displaying elevated profibrotic and inflammatory gene expression levels. Macrophages within the IPFP also undergo significant changes during OA progression. Homeostatic macrophages are involved in immune surveillance and maintaining internal homeostasis in normal conditions. In early OA, macrophages exhibit upregulation of pro-inflammatory cytokines, and as OA advances, markers are further upregulated and associated with pro-inflammatory and tissue remodelling functions. Adipocytes within the IPFP also display notable alterations during OA. In the early stages of OA, adipocytes show increased secretion of pro-inflammatory adipokines. In advanced OA, these cells demonstrate dysfunction, characterised by alterations in lipid metabolism that contribute to the inflammatory environment within the joint. Endothelial cells (EDCs) regulate vascular function and support tissue perfusion. During early OA, EDCs demonstrate increased expression of angiogenesis-related markers. In advanced OA, aberrant angiogenesis and elevated expression of inflammatory markers are evident, further exacerbating the inflammatory environment and contributing to pathological tissue remodelling within the IPFP.

**Table 6:** Infrapatellar fat pad spatiotemporal changes at the micro level in OA progression.

Tissue	Cell Type / Subpopulation		Location	Markers	Function	Early OA	Advanced OA
IPFP [157, 158]	Fibroblasts	Precursor-like	Throughout IPFP	CD26/DPP4, CD10/MME, HLA-C	Aid in tissue repair, shock absorption, and support joint structure	Increased fibrosis markers, inflammatory activation	Advanced differentiation state, higher profibrotic and inflammatory gene expression
		**Differentiated**					
		**Adipogenic**					
		**Pro-inflammatory**					
		**Fibrotic**					
	Macrophages	Homeostatic	Scattered in IPFP	P2RY14, MAN1A1, FGF13	Immune surveillance, maintain tissue homeostasis	Elevated pro-inflammatory cytokines	Increase in pro-inflammatory and tissue remodelling markers
		**Inflammatory**					
		**Tissue Remodeling**					
		**Anti-inflammatory**					
	Adipocytes	Mature Adipocytes	IPFP bulk tissue	CLSTN2, WDPCP, PDE11A	Energy storage, release of adipokines	Increase in pro-inflammatory adipokines	Increased adipocyte dysfunction, changes in lipid metabolism
		**Lipid Metabolic**					
		**Pre-adipocytes**					
		**Stress-responsive**					
	EDCs	Vascular Homeostasis	Vascular structures in IPFP	CADM2, BTNL9, CD36	Regulate vascular function, support tissue perfusion	Increased angiogenesis-related markers	Abnormal angiogenesis, higher expression of inflammatory markers
		**Angiogenic**					
		**Pro-inflammatory**					
		**Barrier Function**					

**BTNL9**: Butyrophilin like 9; **CADM2**: Cell adhesion molecule 2; **CD10/MME**: Cluster of differentiation 10 / membrane metallo-endopeptidase; **CD26/DPP4**: Cluster of differentiation 26 / dipeptidyl peptidase 4; **CD36**: Cluster of differentiation 36; **CLSTN2**: Calsyntenin 2; **EDCs**: Endothelial cells; **FGF13**: Fibroblast growth factor 13; **HLA-C**: Human leukocyte antigen C; **IPFP**: Infrapatellar fat pad; **MAN1A1**: Mannosidase alpha class 1A member 1; **P2RY14**: Purinergic receptor **P2Y14**; **PDE11A**: Phosphodiesterase 11A; **WDPCP**: WD repeat-containing planar cell polarity effector.

### 3.5 Challenges in Combining scRNA-Seq and Spatial Transcriptomic Data in OA Progression

While scRNA-seq and spatial transcriptomics have revolutionised our understanding of cellular heterogeneity and spatial organisation in OA, integrating these two datasets presents significant challenges. Data integration requires sophisticated computational tools to align datasets with differing resolutions and scales. This complexity is exacerbated by sample preparation and processing protocol variations, which can introduce technical biases. Secondly, the high dimensionality of these datasets often necessitates advanced statistical frameworks and machine learning models, which require expertise and computational resources that may not be universally accessible. Lastly, the lack of standardised pipelines for data integration hinders reproducibility and cross-study comparisons. Addressing these challenges will require collaborative efforts to develop robust computational frameworks, standardised protocols, and accessible tools for integrating multi-omics data in OA research.

## 4. Spatiotemporal Development Trajectory in OA from the Molecular Perspective

### 4.1 Genetic and Epigenetic Polymorphism

Genetic and epigenetic factors play a crucial regulatory role in the OA spatiotemporal development trajectory. Genetic variations and epigenetic modifications collectively influence an individual's susceptibility to OA, the rate of disease progression, and the specific characteristics of affected joints. Studies on gene variations, such as collagen type II alpha 1 chain (COL2A1) [159, 160] and growth differentiation factor 5 (GDF5) [161-163], have enabled scientists to identify individuals more prone to OA, determining the age of onset and the severity and progression patterns of the disease. Simultaneously, epigenetic mechanisms, such as DNA methylation, histone modifications, and interactions with non-coding RNA, offer a meticulous gene regulation capability of adjusting to environmental stimuli. These genetic and epigenetic factors significantly impact the pathophysiology of OA by modulating the expression of key catabolic enzymes and inflammatory mediators, driving cartilage degradation and synovial inflammation (Table 7). The detailed review is summarised below:

**Table 7:** Genetic and Epigenetic Factors in OA Progression.

Category	Description	Examples/Key Points	Impact on OA
Genetic Factors	Genetic variations influence susceptibility, progression, and severity of OA.	COL2A1: Collagen type II alpha 1 chain [159, 160]	Genetic variations are linked to OA onset age, severity, and progression patterns. Polymorphisms in key genes like collagen and growth factors identify individuals at higher risk.
		**GDF5: Growth differentiation factor 5 [161-163]**	
		**TLR9: rs187084 polymorphism [164, 165]**	
Polymorphisms in OA-Related Genes	Specific gene polymorphisms contribute to OA risk and severity.	TLR9: Knee and hip OA risk [164, 165]	These genetic polymorphisms influence inflammatory responses and cartilage degradation. Increased risk in knee and hip OA and general OA severity.
		**IL-1β, IL-6: Cytokine-related genes [30]**	
		**MMP-1, MMP-13: Matrix metalloproteinase genes [30]**	
Epigenetic Mechanisms	Epigenetic factors such as DNA methylation, histone modification, and non-coding RNAs regulate gene expression and OA progression.	EZH2: Histone modification leading to miR-138 suppression [170]	Epigenetic regulation modulates inflammatory responses and cartilage metabolism, influencing OA development. It adds a layer of flexibility, adapting to environmental stimuli.
		**DNA Methylation: Changes in collagen genes and inflammatory mediators [171]**	
DNA Methylation	DNA methylation controls gene transcription, contributing to OA pathogenesis.	Hypermethylation of collagen genes leads to reduced collagen synthesis.	Alterations in methylation patterns modify the gene expression associated with cartilage degradation and inflammation, facilitating OA development.
		**Demethylation of inflammatory genes increases their expression [171]**	
Histone Modifications	Histone acetylation/deacetylation and methylation play key roles in OA development.	HDAC2, HDAC4: Regulate inflammation and matrix degradation [30]	Histone modifications regulate inflammatory molecules and matrix-degrading enzymes, affecting cartilage integrity. Inhibiting HDACs may reduce OA-associated inflammation.
		**EZH2: Catalyzes H3K27 methylation [170]**	
Histone Methylation	Histone methylation involves H3K9 and H3K4, influencing gene expression and OA progression.	H3K9: Suppresses gene expression [174]	Methylation patterns in histones regulate OA-related genes, with H3K9 silencing and H3K4 activation influencing OA progression. Histone demethylases play a role in controlling matrix degradation.
		**H3K4: Promotes gene expression [174]**	
		**KDM2A, KDM6A, KDM7A: Histone demethylases upregulated in OA [183]**	
Non-Coding RNAs	Non-coding RNAs (lncRNA, circRNA, miRNA) regulate gene expression, inflammation, and matrix metabolism in OA.	miR-17: Inhibits MMP13 and ADAMTS5 expression [179]	Non-coding RNAs regulate critical pathways in OA. They can inhibit or promote inflammation, cartilage degradation, and OA progression. Elevated miRNA levels can serve as biomarkers.
		**miR-149: Suppresses inflammation via VCAM-1 [180]**	
		**miR-34a-5p: Promotes OA progression [181]**	
**Polymorphisms in Non-Coding RNAs**	Genetic variations in non-coding RNA genes may alter their expression and role in OA.	miR-23a-3p, miR-146a-5p, miR-652-3p: Elevated expression in OA patients [183]	Variations in non-coding RNA genes affect OA progression. Specific miRNAs may serve as diagnostic markers for OA.
**Gene-Environment Interactions**	The combined effect of genetic and environmental factors influences OA development and progression.	Interactions between non-coding RNAs and histone-modifying enzymes (e.g., miR-222 targeting HDAC4) [182]	Gene-environment interactions shape OA pathogenesis, offering opportunities for personalised treatment strategies.

**ADAMTS5**: A disintegrin and metalloproteinase with thrombospondin motifs 5; **COL2A1**: Collagen type II alpha 1 chain; **EZH2**: Enhancer of zeste homolog 2; **GDF5**: Growth differentiation factor 5; **HDAC2**: Histone deacetylase 2; **HDAC4**: Histone deacetylase 4; **H3K4**: Histone 3 lysine 4; **H3K9**: Histone 3 lysine 9; **IL-1β**: Interleukin 1 beta; IL-6: Interleukin 6; **KDM2A**: Lysine demethylase 2A; **KDM6A**: Lysine demethylase 6A; **KDM7A**: Lysine demethylase 7A; **lncRNA**: Long non-coding RNA; **miRNA**: MicroRNA; miR-17: **MicroRNA-17**; miR-23a-3p: MicroRNA-23a-3p; **miR-34a-5p**: MicroRNA-34a-5p; **miR-149**: MicroRNA-149; miR-146a-5p: MicroRNA-146a-5p; **miR-652-3p**: MicroRNA-652-3p; **MMP13**: Matrix metalloproteinase 13; **MMP-1**: Matrix metalloproteinase 1; **OA**: Osteoarthritis; Polymorphisms: Genetic variations affecting susceptibility to disease; **SOST**: Sclerostin; **TLR9**: Toll-like receptor 9; **VCAM-1**: Vascular cell adhesion molecule 1.

Genetic polymorphisms are significant in the pathogenesis of OA. Polymorphisms in specific genes are linked to the risk and severity of OA. For instance, Toll-like receptor 9 (TLR9) gene rs187084 polymorphism is linked to a heightened risk of knee and hip OA [164, 165]. In contrast, Toll-like receptor 10 (TLR10) gene rs11096957 polymorphism is linked to increased hip OA risk and severity [166]. The RAGE gene 82G/S polymorphism is also associated with an increased risk of knee OA [167]. Polymorphisms in cytokine and enzyme-related genes, such as interleukin-1 beta (IL-1β), Interleukin 1 Receptor Antagonist (IL-1RN), Interleukin 18 (IL-18), IL-6, MMP-1, Matrix Metallopeptidase 13 (MMP-13), and ADAM Metallopeptidase with Thrombospondin Type 1 Motif 14 (ADAMTS14), are also associated with increased OA risk and severity [30]. Other genes like B-cell Lymphoma 2 (BCL-2) [168], C-X-C Motif Chemokine Ligand 16 (CXCL16) [168], ITLN1/omentin [35], DOT1L [35], and matrix Gla protein [169] also show increased risk association with OA.

Epigenetic regulation, such as histone modifications, can influence gene expression, thereby regulating inflammatory responses and cartilage metabolism, contributing to the development of OA. For example, the enhancer of zeste 2 polycomb repressive complex 2 subunit (EZH2) promotes OA progression by methylating histone H3 lysine 27 (H3K27) to suppress miR-138. It can also regulate matrix metalloproteinases and inflammatory factors independently of its enzymatic activity [170]. The significant function of epigenetic mechanisms in the development of OA calls for additional research, such as exploring the interplays between genetic and epigenetic factors.

DNA methylation is a crucial epigenetic regulatory mechanism playing a significant role in OA pathogenesis. In OA patients, genes encoding collagen molecules undergo hypermethylation, inhibiting their transcription and promoting OA development. Conversely, demethylation of promoter regions of inflammatory mediator genes leads to their increased expression [171]. Differentially methylated enhancer regions between OA patients and normal individuals have also been identified. Therefore, DNA methylation regulates OA-related gene transcription, contributing significantly to OA pathogenesis.

Histone modifications also play essential roles in OA pathogenesis. Histone acetylation and deacetylation are critical epigenetic regulatory mechanisms. Histone deacetylase 2 (HDAC2) and Histone deacetylase 4 (HDAC4) regulate molecules related to inflammation and matrix degradation in OA [30]. Histone deacetylase inhibitors were shown to regulate immune response, stabilise mitochondria, and stabilise microtubules, which play a protective role in OA progression [172, 173]. Histone methylation is also involved in OA pathogenesis. Histone H3 lysine 9 (H3K9) methylation is typically associated with gene expression suppression, while Histone H3 lysine 4 (H3K4) methylation promotes gene expression [174]. EZH2, a histone methyltransferase, catalyses the methylation of histone H3 on lysine 27 (H3K27), which is usually associated with transcriptional repression [170]. However, EZH2 may also influence gene expression through other mechanisms, such as DNA methylation and hydroxymethylation [175]. Furthermore, upregulation of various histone demethylases, such as lysine-specific demethylase 2A (KDM2A), lysine-specific demethylase 6A (KDM6A), and lysine-specific demethylase 7A (KDM7A), has been observed in OA patients. Inhibiting Histone Nε-methyl lysine demethylases (KDM2/7) can increase lysine 79 of histone H3 (H3K79) methylation, inhibiting cartilage damage [176].

Non-coding RNAs, including long non-coding RNAs (lncRNA), circular RNAs (circRNA), and small non-coding RNAs (sncRNA) like microRNAs (miRNA), small interfering RNAs (siRNA), and piwi-interacting RNAs (piRNA), are crucial genetic regulatory factors in OA [177]. These non-coding RNAs regulate gene expression through various mechanisms [178]. For instance, miRNAs can post-transcriptionally inhibit gene expression, while lncRNAs and circRNAs can act as competing endogenous RNAs or sponges. Research has shown that non-coding RNAs play significant roles in the progression or inhibition of OA. For example, miR-17 can inhibit the expression of Nitric oxide synthase 2 (Nos2), ADAM Metallopeptidase With Thrombospondin Type 1 Motif 5 (ADAMTS5), MMP-3, and MMP-13, protecting cartilage [179]. miR-149 can inhibit inflammation by suppressing Vascular cell adhesion protein 1 (VCAM-1) [180], while miR-34a-5p can reduce Collagen Type II Alpha 1 Chain (COL2A1) and aggrecan expression and increase MMP-13 and ADAMTS5 expression, promoting OA progression [181]. Non-coding RNAs interact with histone-modifying enzymes; for instance, miR-222 targets HDAC4, inhibiting MMP-13 expression [182]. Polymorphisms and methylation of non-coding RNA genes can also affect their expression and function, contributing to OA pathogenesis. Specific miRNAs, such as miR-23a-3p, miR-146a-5p, and miR-652-3p, show elevated expression levels in OA patients, potentially serving as diagnostic markers for OA [183]. Consequently, non-coding RNAs are essential genetic regulatory elements that participate in the pathogenesis of OA via diverse mechanisms. The above genetic and epigenetic factors could serve as clinical biomarkers to distinguish high-risk individuals for OA. However, the above spatiotemporal changes of the genetic and epigenetic change remain to be discovered, especially for the tissue-specific change. Conflicting study results might be attributed to sample size or population differences. Some genetic variants might be less common in specific populations, necessitating large-scale studies across diverse groups to evaluate their precise impact. A comprehensive understanding of these factors' spatiotemporal variations across different populations and individuals offers new perspectives for precision medicine, aiding in developing personalised therapeutic strategies tailored to specific genetic and epigenetic profiles, thus enhancing the efficacy of OA treatments.

### 4.2 Biochemical Pathways

The biochemical landscape of OA is dominated by a cascade of molecular pathways that contribute significantly to the cartilage degradation and inflammation characteristic of the disease even before the occurrence of visible radiographic changes. In a cohort of 200 women, 24 serum protein biomarkers were identified to predict knee OA before radiographic abnormalities. The predictions based on these blood biomarkers outperform traditional predictions based on age and BMI (AUC 51%) or knee pain (AUC 57%). Among them, the majority (58%) also predict the progression of knee OA. This discovery implies that the molecular pathophysiology beneath incident OA and progressive knee OA is similar, thus uncovering the presence of a pathophysiological "OA continuum". [64].

Traditional biochemical players in these pathways include cytokines such as Interleukin-1 beta (IL-1β) and tumour necrosis factor-alpha (TNF-α), which initiate and perpetuate inflammatory processes within joint tissues [184, 185]. Alongside these cytokines, MMPs are crucial for degenerating extracellular matrix components, directly leading to cartilage erosion [186]. These biochemical mediators do not act uniformly across all affected joints or throughout all stages of OA. Instead, their expression and activity can vary significantly, contributing to the temporal dynamics of disease progression—where early stages may show mild symptoms that escalate as the biochemical activity intensifies. Spatial variations are also evident, as different joints may exhibit distinct biochemical signatures depending on the mechanical stresses they endure and their biological responses. Understanding these spatiotemporal changes in biochemical pathways is essential for developing targeted therapeutic interventions that can potentially halt or reverse the progression of OA at various stages and in specific joints.

With the emergence of more research cohorts in OA research, researchers started to utilise multiple biomarkers capable of tracking the temporal changes of OA to classify the endotypes. Recently, a study employed machine learning techniques to analyse the biochemical marker data from the IMI-APPROACH cohort to identify different subtypes of OA [67]. This innovative and forward-looking research method provides new ideas and means for the precise classification of OA. The IMI-APPROACH is a prospective cohort study that includes 297 knee OA patients who meet the American College of Rheumatology classification criteria. These patients were pre-selected from existing cohorts through a machine learning model that indicated a higher possibility of joint space width reduction and/or the progression of knee pain. Among the 433 OA patients screened from 5 centres, 297 were finally included because they were most likely to experience pain and/or structural progression during the 2-year follow-up period. This screening process is rigorous and scientific, ensuring the pertinence and representativeness of the research subjects. Three main subtypes of OA were identified by analysing 16 biochemical markers related to joint tissue metabolism in the cohort. Low tissue metabolism, low repair ability, and cartilage/bone metabolism levels characterise the C1 subtype. The C2 subtype is associated with structural damage, featuring high bone formation/resorption and cartilage degradation. The C3 subtype involves systemic inflammation, including joint tissue degradation, inflammation, and cartilage degradation. This classification method based on biomarkers provides important clues for a deeper understanding of the pathogenesis of OA [67]. Significant differences exist among these three subtypes regarding clinical characteristics and disease progression. The C1 subtype has the highest proportion of patients without disease progression, indicating the least degree of disease progression. The C2 subtype is mainly related to the longitudinal structural changes of joints over time. The C3 subtype is linked to persistent or worsening pain symptoms. This finding is significant for clinical treatment and helps doctors formulate personalised treatment plans for different subtypes. The biomarker-based, data-driven subtype identification method may significantly value patient stratification in OA clinical trials and contribute to implementing precision medicine strategies. It can be foreseen that with the continuous progress of technology and the deepening of research, this method will play an increasingly important role in diagnosing and treating OA [67, 187].

Meanwhile, data-driven techniques for OA predictions have surged in recent years, which indicates that multiple molecular changes happen before the radiographic changes. Due to the construction of various databases containing multi-omic and radiographic data, some omics biomarkers have replaced traditional clinical risk markers, with several proving to be more predictive of OA risk than BMI. Another latest study incorporated clinical, lifestyle and biomarker data using the UK Biobank database [49]. They divided OA into 14 subgroups using interpretable machine learning [49], and they found out that key gene and signalling pathways, including growth differentiation factor 5 (GDF5) and transforming growth factor beta (TFG-β) signalling pathways, play an essential role in OA progression. Cartilage acidic protein 1 (CRTAC1) emerged as the most predictive protein, previously identified as an OA risk biomarker [49, 188-190]. CRTAC1 is linked to OA diagnosis in various joints and correlates with the severity of OA [188-190]. Pro-inflammatory cytokines are believed to upregulate CRTAC1 in OA-affected joints [191]. Another significant biomarker, collagen type IX alpha 1 chain (COL9A1), predicts OA diagnosis and has genetic and epigenetic associations with OA [191-195]. Mutations in COL9A1 are also linked to multiple epiphyseal dysplasia, a hereditary condition marked by early-onset OA [191]. Additionally, actin alpha 2 (ACTA2) has been associated with specific OA subgroups and smooth muscle cell clusters in the OA synovium [196]. Finally, the ectodysplasin A2 receptor (EDA2R), another predictive protein, has been linked to TNF-mediated inflammation in RA [197]. The above studies provide a novel perspective for OA's precision prevention and treatment development.

### 4.3 Metabolic Changes

Metabolic changes play a crucial role in the spatiotemporal development trajectories of OA from a molecular perspective. During the early stages of OA, there is a notable shift in chondrocyte metabolism, characterised by increased glycolysis [198] and a corresponding decrease in oxidative phosphorylation [199]. This metabolic reprogramming is driven by hypoxic conditions within the cartilage, leading to upregulation of hypoxia-inducible factor 1-alpha (HIF-1α). As OA progresses, the altered metabolic state of chondrocytes results in the accumulation of lactate and other metabolic by-products, contributing to an acidic microenvironment that exacerbates cartilage degradation [200]. Furthermore, inflammatory cytokines such as IL-1β and TNF-α amplify these metabolic disruptions by inducing nitric oxide synthase (NOS) activity, further impairing mitochondrial function and energy production [201, 202]. The dysregulation of anabolic and catabolic gene expression also influences the catabolic processes, including the suppression of collagen type II and aggrecan synthesis and the upregulation of MMPs and aggrecanases. In advanced stages of OA, synovial inflammation and subchondral bone sclerosis add complexity to the metabolic landscape, promoting a feedback loop that perpetuates joint degeneration [203]. Understanding these metabolic changes at various stages of OA development is essential for identifying potential therapeutic targets to restore metabolic balance and halt disease progression.

**Normal**: A novel study used matrix-assisted laser desorption/ionisation Fourier transform ion cyclotron resonance mass spectrometry (MALDI-FT-ICR-MS) imaging experiments on human knee OA cartilage for technical validation [204]. They detected spatial differences in the distribution of metabolites such as adenosine triphosphate (ATP), adenosine diphosphate (ADP), and adenosine monophosphate (AMP). These nucleotide metabolites were mainly concentrated in the superficial zone of the cartilage rather than in the deeper layers. The authors hypothesised that a particular situation could be explained by the fact that the superficial part of the cartilage has direct contact with the synovial fluid and that it is the only region where cartilage progenitor cells have been identified.

**OA**: Recent advances in the molecular changes of OA from a spatiotemporal perspective mainly focus on spatial metabolomics and lipidomics. A pivotal study examined the femoral heads of patients in advanced OA (grades III-IV), revealing that metabolic phenotypes vary according to the stage of the disease and the specific joint regions affected. Advanced analysis of cartilage and subchondral bone samples from quadrants of the femoral head identified critical metabolic pathways driving these variations. These pathways include lipid metabolism, amino acid metabolism, nucleotide metabolism, and glucose metabolism. Notably, glycosaminoglycan degradation and amino acid metabolism were predominantly concentrated in the upper regions of the femoral head, suggesting that metabolic disruptions in advanced OA are anatomically and pathologically heterogeneous [205]. OA synovium's lipidomic profile has also exhibited distinctive characteristics compared to healthy synovium and other inflammatory arthropathies such as RA and PsA. In OA synovium, elevated levels of phosphatidylcholine, fatty acids, and lysophosphatidic acid contrasted with reduced levels of lysophosphatidylcholine. Furthermore, spatial distribution analysis revealed that specific glycerol-phospholipid profiles were associated with hypertrophy, inflammation, and vascularisation of the synovium. Interestingly, OA tissues exhibited lower levels of phosphatidylethanolamine plasmalogens compared to RA and PsA counterparts. These findings emphasise the unique lipidomic signature of OA synovium and its potential utility in diagnostic and therapeutic applications [206]. These investigations provide invaluable insights into OA's complex metabolic and lipidomic adaptations. The findings may shed light on developing precise diagnostic tools and targeted interventions, paving the way for enhanced patient care and improved disease management strategies.

### 4.4 Challenges in Combining Existing Spatial-Omics Data in OA Progression

Combining spatial-omics data in OA progression, including spatial transcriptomics, proteomics, metabolomics, elemental mapping, and mechanical mapping, presents several challenges. Each omics layer provides unique insights—transcriptomics reveals gene expression, proteomics shows protein localisation and metabolomics tracks biochemical processes. Integrating these diverse datasets while maintaining spatial accuracy is challenging. The overlap between molecular data from different layers is not always perfect, making it challenging to correlate findings across platforms. The volume of spatial transcriptomics and proteomics data can be overwhelming, requiring advanced computational tools. Developing algorithms to reduce dimensionality while preserving essential biological information is crucial but complex. Managing these large-scale datasets demands efficient storage, processing, and analysis methods. Different spatial-omics techniques have varying levels of spatial resolution and sensitivity. While spatial transcriptomics offers high-resolution gene expression data, proteomics and metabolomics may have lower spatial resolution, making capturing fine-scale changes in molecular interactions difficult. Similarly, mechanical mapping, which assesses tissue stiffness, lacks the sensitivity for early-stage OA detection. Integrating molecular data with mechanical properties (e.g., cartilage stiffness) requires sophisticated multivariate analysis. OA exhibits patient-specific progression patterns, so developing machine learning models to correlate these data types and predict disease trajectories should be further developed. Existing methods often fall short in accounting for OA’s complex biological and mechanical features. Integrating these diverse data types requires collaboration across multiple disciplines, including biology, chemistry, physics, and computational science. This interdisciplinary effort is necessary for designing experiments, interpreting complex data, and developing tools that can handle multi-omics data effectively. In summary, the main challenges in combining spatial-omics data for OA research involve data integration, scaling, resolution, sensitivity, and variability. Overcoming these barriers requires advanced computational methods, interdisciplinary teamwork, and ongoing technological improvements.

## 5. Integrative Insights and Therapeutic Implications

### 5.1 Novel Technologies and Methodology in OA Spatiotemporal Changes

As the understanding of OA evolves, emerging tools and technologies significantly enhance the study of OA’s spatiotemperal dynamics. Among those, three crucial techniques are needed to understand OA progression: high-resolution imaging techniques, quantitative biomarker surveillance and artificial intelligence/machine learning.

High-resolution imaging techniques, featured by spatial transcriptomics and spatial proteomics, are complementary fields that study the spatial organisation of molecular components within biological systems [24, 207]. Spatial transcriptomics maps gene expression by localising RNA molecules within tissue architecture, using methods like in situ hybridisation and spatial barcoding to uncover patterns of gene activity and tissue heterogeneity. Spatial proteomics focuses on protein localisation and interactions, employing advanced imaging and mass spectrometry techniques to investigate cellular functions and disease mechanisms. Together, these fields integrate molecular and spatial data, providing a comprehensive understanding of how the spatial organisation of transcripts and proteins influences biological processes and disease. With spatial proteomics and spatial transcriptomics won as Nature Method of the Year 2024 and 2020, respectively [24, 207], these up-to-date techniques, alongside spatial metabolomics, provide a probability for researchers to understand OA progression. Technical advances have been advancing in two directions: to reach ultra-high-resolution to reach a subcellular spatial resolution and to correlate with multi-omics data in situ. Higher resolutions are mainly achieved by coupling fluorophore labelling using optical scanning microscopy or ToF-SIM, a mass spectrometry imaging-based system. Advancements in technology have opened new avenues for understanding the progression of OA by linking spatial proteomics with transcriptomics. In contrast to traditional clinical imaging techniques, newly developed multi-omics imaging platforms now offer comprehensive solutions for analysing spatial transcriptomics and spatial proteomics and famous instruments include CODEX (Akoya Bioscience), Stereo-seq (Beijing Genomics Institute), Visium (10× Genomics), GeoMx DSP (Nanostring), CosMx SMI (Nanostring), MERFISH (Vizgen), MultiOmyx (NeoGenomics), Discovery Ultra/Opal multiplex IHC (Roche/Akoya Bioscience), Hyperion (Fluidigm), and MIBIscope (IONpath) [207]. Other imaging techniques, like hyperspectral imaging techniques, elemental mapping, and function imaging, can also help reveal the spatiotemporal changes of OA [8]. To conclude, novel methodologies, models and instruments offer unparalleled insights into OA's spatiotemperal dynamics, driving precision diagnostics and innovative therapies for improved patient outcomes.

Although consecutive histopathological tracing of the human knee joint in vivo is still tricky, the dynamic tracing for OA progression in an animal model has shed light on OA progression. The practice hasn’t been utilised in OA changes yet, but the mouse embryo development as the spatiotemporal transcriptomic modification patterns have been verified using various techniques, including Slide-seq and the three-dimensional reconstruction and visualisation tool (sc3D) [208], Stereo-seq using DNA nanoball [209] and sequential fluorescence in situ hybridisation (seqFISH) [210].

Artificial Intelligence (AI) holds transformative potential in OA research, particularly in three key areas: integrating data across different scales, automating the annotation of genes or proteins, and uncovering risk factors or biomarkers through data-driven approaches. Machine learning (ML) algorithms can combine spatial multi-omics data with clinical and biochemical indicators, bridging the gap between laboratory research and clinical application. By leveraging ML in spatial proteomics, researchers can improve image-based analyses, enabling precise and efficient identification of proteomic signatures within joint tissues [211]. However, ML heavily relies on annotation libraries derived from in-silico experimental data, meaning its effectiveness depends on the availability of highly accurate, tissue-specific proteomic data. Unfortunately, current efforts to construct and standardise proteomics databases for cartilage remain inadequate and require further development. While these advancements in technology and methodology offer promising opportunities to enhance OA diagnosis and treatment, their successful implementation in clinical practice will depend on overcoming significant logistical and technical challenges.

### 5.2 Emerging Research Direction and Theoretical Frame for OA Aetiology

**Figure 3.**
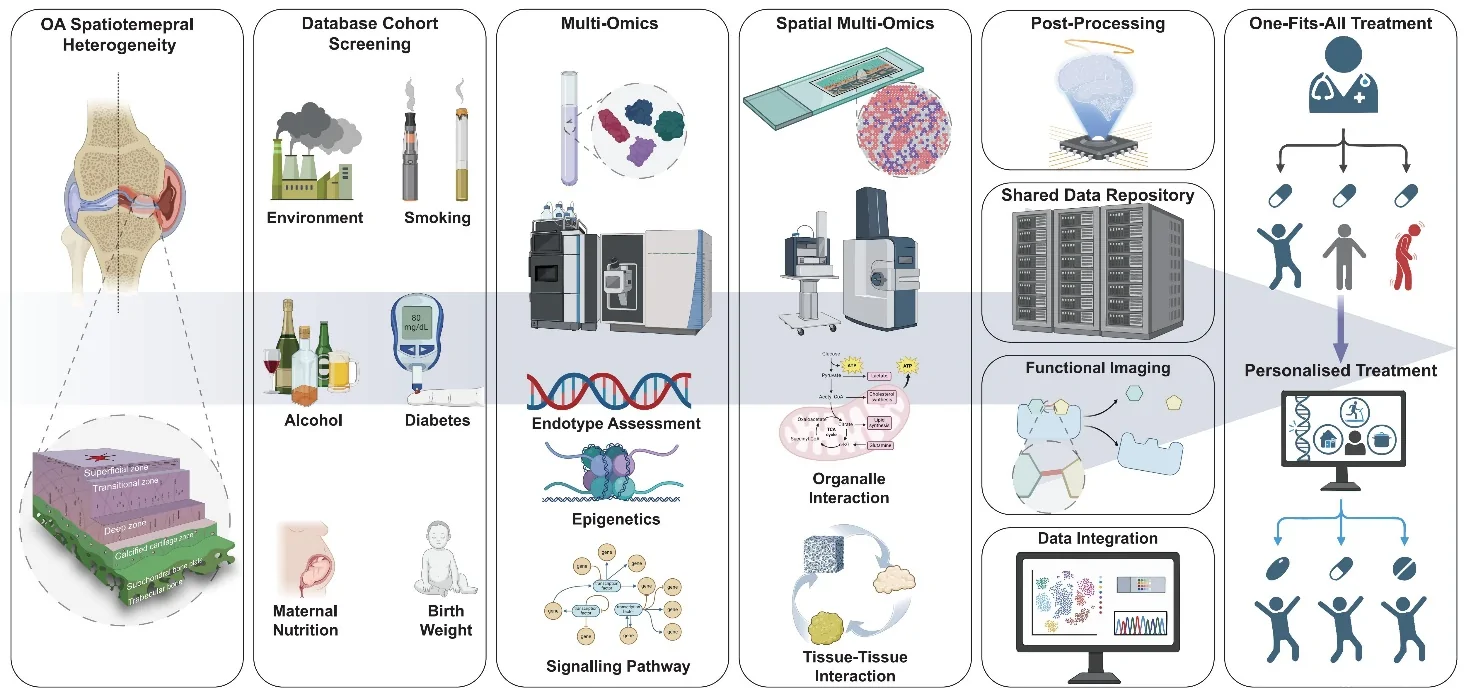
**Future integrated approach to investigating phenotypes and endotypes of OA for precision treatment**. Future understanding requires addressing the spatiotemporal complexity of OA to advance personalised treatment strategies. Due to complex interplay, OA’s spatiotemporal heterogeneity highlights the tissue-related differences in reacting to OA. Database cohort screening set the foundation to identify key risk factors that influence OA onset and progression, such as environmental pollutants, smoking, alcohol consumption, diabetes, maternal nutrition, and birth weight. Multi-omics approaches, such as endotype assessment, epigenetics, and the analysis of signalling pathways, provide insights into the molecular mechanisms driving disease variability. Spatial multi-omics techniques further explore organelle interactions and tissue-tissue dynamics, revealing the spatial organisation of disease processes. Advanced data processing is supported by shared repositories and computational tools that enable functional imaging and the integration of diverse datasets. The culmination of this framework allows for the shift from generic "one-size-fits-all" treatment approaches to personalised therapies tailored to individual patient profiles, incorporating genetic, molecular, and lifestyle factors to optimise care and outcomes. This integrative approach highlights the importance of precision medicine in OA management. The figure was created with Biorender.com

Regarding research models, four key aspects of OA progression are crucial: organelle-organelle, cell-cell, tissue-tissue communication, and cross-scale interactions [212]. Multi-scale models integrating data across molecular, cellular, tissue, and organ levels are essential for understanding OA progression. Despite significant advances, cross-scale investigations remain underdeveloped. Key areas for exploration include (1) correlating local histological changes with systemic biomarker shifts, (2) developing robust post-processing techniques for large-scale imaging and phenotype data, (3) linking spatial endotype changes in local tissues with clinical phenotypes and imaging, and (4) associating developmental gene polymorphisms with late-stage OA (Fig. 3).

A critical challenge in OA research is translating histopathological knowledge into clinical practice. One promising pathway involves integrating big and multi-omics data, such as the UK Biobank. For instance, MILTON's machine learning tool predicts 3,213 diseases using biomarkers and longitudinal health records, outperforming polygenic risk scores and identifying novel gene-disease associations [213]. Similarly, "sc-linker" connects GWAS findings with specific cell types and processes by integrating scRNA-seq data, epigenomic SNP-to-gene maps, and GWAS summary statistics [214]. These advances may inspire future investigation of data-driven approaches to enhance genetic research and personalised OA care.

Another challenge in understanding OA's heterogeneity requires distinguishing phenotypes—observable traits from clinical assessments—and endotypes, which reflect underlying pathobiological mechanisms like inflammation or biomechanical stress [67, 187, 215-218]. This distinction highlights that different mechanistic pathways may result in similar clinical presentations but require tailored therapeutic strategies. Although separately investigated, the combination of spatiotemporal analysis in the OA field remains to be investigated, which paved the way for future personalised interventions [219].

Despite identifying novel cell subtypes through scRNA-seq and multi-omics imaging, their roles in OA progression remain unclear. For example, while infants can regenerate cartilage, this ability diminishes with growth. Whether changes or manipulation to specific cell subtypes contribute to loss of regeneration remains to be determined. OA's biomechanical nature also suggests that cell interactions in different environments may vary, presenting opportunities to identify vulnerable subtypes and develop targeted therapies. These unresolved questions highlight the complexity of modelling OA's molecular and cellular mechanisms. Advancing our understanding of these interactions will require collaborative efforts across disciplines to address the temporal and spatial dynamics of tissue degeneration.

### 5.3 Precision Medicine Targeting Spatiotemporal Risk Factors of OA

Emerging therapies in OA are increasingly informed by our evolving understanding of the disease's spatiotemporal dynamics. Recent advances in pharmacological treatments focus on targeting specific subtypes of OA based on spatiotemporal subtypes implicated in the progressive degeneration of joint tissues.

For example, metabolic regulative drugs have been proven to have life-changing benefits in OA [220]. Semaglutide is a Glucagon-like peptide-1 (GLP-1) agonist approved by the FDA for treating obesity. In the latest study involving a 68-week randomised trial, once-weekly injectable semaglutide significantly reduced body weight and knee OA pain compared to placebo in obese participants with moderate-to-severe pain. At the same time, adverse events were similar between groups, proving the efficacy of semaglutide on obese patients with knee OA [221]. NAD^+^ is another hot topic in metabolic improvement. The Sirtuin (SIRT) family consists of nicotinamide adenine dinucleotide (NAD^+^)-dependent protein deacetylases that play an active role in regulating chondrocyte functions during the pathophysiology of OA. This family is crucial for preventing cartilage aging and degradation by maintaining extracellular matrix (ECM) homeostasis, regulating chondrocyte metabolism, and inhibiting chondrocyte apoptosis and autophagy through their deacetylation activity. [222, 223]. Mouse models have demonstrated that inhibition of CD38, an enzyme of NAD^+^, may serve as a promising therapeutic strategy for OA treatment by reducing cartilage degradation, synovial inflammation, and associated pain following joint injury [224]. Other FDA-approved metabolic manipulation medicines and therapies, like statins [225, 226], metformin [227], caloric restriction [228], minimally invasive arthroscopy and autologous micro-fragmented adipose tissue transplantation [229], can also be seen as future strategies.

Meanwhile, multiple studies have proved that anti-aging strategies benefit OA [230, 231]. Key regulators in aging, including stem cell therapy [232], senescent cell elimination [233], autophagy enhancement [234], physical exercise [235], cellular reprogramming [236] and telomere reactivation [237], Parabiosis (Blood Exchange) [238] and other techniques have also raised researchers' interest and proven to be promising in OA. Despite advancements in therapy, the foundational approach of spatiotemporal phenotyping and clustering remains unexplored. Existing precision medicine strategies predominantly involve systemic drug treatments, often resulting in unnecessary side effects. Future research could illuminate methods for the targeted enrichment of medicinal compounds at specific sites, potentially halting or reversing the progression of OA.

## 6. Conclusion

In summary, incorporating spatiotemporal dynamics offers a holistic view for exploring the interactions between tissues in OA research. This interdisciplinary strategy could improve our knowledge of this suffering disease that impacts millions globally. A deeper understanding of the fundamental mechanisms underlying OA in a spatiotemporal manner provides essential evidence for developing effective DMOADs. This advancement can improve treatment options and, subsequently, the quality of life for individuals affected by this debilitating condition.
